# Investigating the role of EFhd2 protein in modulating tau pathology in a tauopathy mouse model

**DOI:** 10.3389/fnmol.2026.1833887

**Published:** 2026-08-12

**Authors:** Ahlam S. Soliman, Andrew Umstead, Irving E. Vega

**Affiliations:** 1 Department of Translational Neuroscience, College of Human Medicine, Michigan State University, Grand Rapids, MI, United States; 2 Neuroscience Program, Michigan State University, East Lansing, MI, United States; 3 Integrated Mass Spectrometry Unit, College of Human Medicine, Grand Rapids, MI, United States; 4 Hauenstein Neuroscience Center, Mercy Health Saint Mary’s, Grand Rapids, MI, United States; 5 Michigan Alzheimer’s Disease Research Center, University of Michigan, Ann Arbor, MI, United States; 6 Department of Neurology, University of Michigan, Ann Arbor, MI, United States

**Keywords:** EFhd2, mouse model, neurofibrillary tangles, oligomers, tau, tauopathies

## Abstract

Accumulation of abnormally aggregated tau is the main pathological hallmark in tauopathies, including Alzheimer’s disease (AD). Increasing evidence suggests that pretangle oligomeric tau aggregates exert neurotoxicity, while neurofibrillary tangles (NFTs) may represent less toxic structures that possibly delay cellular demise. A multitude of *in vitro* studies have endorsed the low propensity of tau protein to aggregate without an external aggregation inducer. Hence, studying tau-interacting proteins has garnered research attention in the last decade as potential contributors to pathological tau aggregation. We identified EF-Hand Domain Family Member D2 (EFhd2) protein as a tau-associated protein in JNPL3 mouse model and postmortem tauopathies tissues. Recently, we have shown that EFhd2 interacts with tau filaments *in vitro*, promoting the formation of large unique aggregated structures. Based on these findings and others, we hypothesized that EFhd2 might contribute to the formation of tau aggregates *in vivo*. To test this hypothesis, we examined the impact of deleting the *Efhd2* gene on the progressive pathological phenotype and neuropathological changes in Tau_*P301L*_-expressing mice. The results indicated only modest, sex-specific differences in lifespan associated with *Efhd2* deletion in Tau_*P301L*_ mice. Independent of *p*-values, *Efhd2* deletion also showed a medium-to-large main effect in reducing cortical pSer422 and PHF1 tau levels in aged mice, consistent with attenuated late-stage tau aggregation. Moreover, *Efhd2* deletion showed effect-size–based trends toward higher Alz50 staining, suggesting an increase in early pathological tau conformations. These findings indicate that *Efhd2* deletion is associated with a shift in tau species toward earlier conformational states alongside reduced markers of later-stage aggregation. The observed modest effects do not establish a direct mechanistic role for EFhd2 in tau aggregation *in vivo*. Rather, they suggest that EFhd2 may function as a modulatory component within a broader protein network influencing tau pathology. Future studies will determine to the extent to which these effects arise from direct modulation of tau aggregation, altered cellular pathways, or interactions within the EFhd2-associated protein network.

## Introduction

Tauopathies encompass highly diverse neurodegenerative disorders, including Alzheimer’s disease (AD), frontotemporal dementia (FTD), and progressive supranuclear palsy (PSP), and the major pathological hallmark thereof is aberrant aggregates of tau protein (Chung et al., 2021; Sexton et al., 2022). In fact, the major differences among tauopathies largely consist in affected brain regions, cellular inclusions, tau aggregates, and clinical presentation (Chung et al., 2021; Sexton et al., 2022). However, they all converge on the abnormal transformation of tau from a highly dynamic protein to a static protein with a great tendency for self-aggregation (Wang and Mandelkow, 2016; Chung et al., 2021). It has become clear that abnormal tau aggregation follows a similar trajectory in all tauopathies (Kuret et al., 2005; Wang and Mandelkow, 2016; Chung et al., 2021; Limorenko and Lashuel, 2022). Abnormal conformational changes of tau promote the formation of early dimers, trimers, and oligomers. Then these oligomeric species further aggregate to tau filaments and fibrils, leading to ultimate high molecular weight aggregates e.g., neurofibrillary tangles (NFTs) in AD (Kuret et al., 2005; Chung et al., 2021; Limorenko and Lashuel, 2022). Evolving insights deem the early pretangle oligomeric aggregates as the true perpetrator, causing neurotoxicity and neurodegeneration (Berger et al., 2007; Brunden et al., 2008; Spires-Jones et al., 2009; Kayed, 2010; Lasagna-Reeves et al., 2011, 2012; Patterson et al., 2011). On the other hand, the formation of neurofibrillary tangles could represent a less toxic response that possibly delays cellular demise (Gomez-Isla et al., 1997; Morsch et al., 1999; Wittmann et al., 2001; Santacruz et al., 2005; Spires et al., 2006; Sydow et al., 2011; Cowan and Mudher, 2013; Kuchibhotla et al., 2014). Therefore, years of extensive research have been dedicated to unraveling the molecular mechanisms that govern abnormal tau conformations and aggregation.

In inherited tauopathies, such as frontotemporal dementia and parkinsonism linked to chromosome 17 (FTDP-17), mutations of *MAPT* gene (that encodes tau) predispose tau to adopt aberrant conformations, leading to the formation of aggregates (Hutton et al., 1998; Wang and Mandelkow, 2016; Strang et al., 2019; Kanaan et al., 2020). In this vein, transgenic tauopathy models that overexpress human mutant tau exhibit progressive pathological aggregation and neurodegeneration modeling some aspects of human diseases. However, in sporadic tauopathies, such as AD and corticobasal degeneration (CBD), with no autosomal mutations, scientists have proposed some factors that might lead to tau pathology. For instance, tau undergoes a large array of post-translational modifications (PTMs). The imbalance in regulating these PTMs disrupts tau structure and promotes its aggregation (Kanaan et al., 2011; Tiernan et al., 2016; Wang and Mandelkow, 2016). A recent surge of interest has been drawn to study tau-interacting proteins as another molecular factor that could impact pathological tau accumulation in tauopathies (Kavanagh et al., 2022; Prikas et al., 2022; Tracy et al., 2022; Betters et al., 2023; Griffin et al., 2023; Younas et al., 2024).

Prior research has demonstrated the link between the relatively novel protein EF-Hand Domain Family Member D2 (EFhd2) and a number of neurological disorders and neurodegenerative diseases (Martins-de-Souza et al., 2009, 2010; Kekesi et al., 2012; Vega, 2016; Mielenz et al., 2018; Broniarczyk-Czarniak and Gałecki, 2019; Kogias et al., 2020). Our group has primarily shown the association of EFhd2 and pathological changes in tauopathies using animal models and human brains. Pioneering structural studies of EFhd2 protein determined three major domains: N-terminus, calcium-binding motif, and coiled-coil domain. The N-terminus domain includes 6–9 alanine residues forming a PolyA tail and a low-complexity flexible region (Avramidou et al., 2007; Ferrer-Acosta et al., 2013a; Kogias et al., 2019). Consistently, initial studies suggested that EFhd2, due to its dynamicity, does not adopt a well-defined tertiary structure (Dyson and Wright, 2005; Ferrer-Acosta et al., 2013a). In fact, this feature characterizes a large group of neurodegeneration-linked proteins, such as tau, α-Synuclein, and TDP-43 (Avila et al., 2016; Lim et al., 2016; Bisi et al., 2021). In addition, the coiled-coil domain (C-C) at the C-terminus mediates EFhd2 self-oligomerization and protein-protein interactions, thereby classifying EFhd2 as an amyloid protein (Liu et al., 2006; Ferrer-Acosta et al., 2013a,b; Park et al., 2017; Szczepaniak et al., 2021). Furthermore, we have shown that EFhd2 phase separates *in vitro* in the presence of a crowding agent into static solid-like structures (Vega et al., 2019). In the presence of calcium, these structures transform into more dynamic droplets. Regardless of the presence of calcium, C-C domain seemed necessary for phase separation of EFhd2, and not the N-terminus (Vega et al., 2019).

EFhd2 exhibits a widespread expression in the body with the highest abundance in the central nervous system (CNS) (Avramidou et al., 2007; Vega et al., 2008). In addition, EFhd2 expression is not homogeneous in the brain. In particular, a higher expression levels of EFhd2 were reported in the cortex, limbic system, and basal ganglia, whereas it exists at lower levels in brains stem and cerebellum (Vega et al., 2008; Purohit et al., 2014). EFhd2 is linked to disparate biological processes, such as cytoskeleton organization, vesicle trafficking, and protein regulation (Soliman et al., 2021). Still, the mechanisms by which EFhd2 could modulate these pathways remain under ongoing research.

We identified a progressive accumulation of EFhd2 with pathological tau aggregates in an age-dependent manner (Vega et al., 2008). Furthermore, EFhd2 exclusively co-purified with sarkosyl-insoluble pathological tau species extracted from animals with a severe behavioral phenotype, whereas EFhd2 was not detected with tau in younger mice. These findings were corroborated in postmortem tauopathies brains of AD and FTD (Vega et al., 2008; Ferrer-Acosta et al., 2013b). Along similar lines, EFhd2 and PHF1 tau (phosphorylated tau at Ser396/404) co-localized in the somatodendritic compartment in the frontal cortex of AD (Ferrer-Acosta et al., 2013b). Moreover, we identified higher EFhd2 levels in AD brains compared to controls. A recent study proposed that this increase in EFhd2 levels in AD is due to the downregulation of miR-126a, a microRNA that regulates EFhd2 expression in the brain (Ferrer-Acosta et al., 2013b; Xue et al., 2022).

Given these findings, we speculated that EFhd2 contributes to the biogenesis of pathological tau aggregation. We tested this hypothesis by measuring thioflavin S (ThS) after incubating EFhd2 with tau’s microtubule-binding repeat K19 fragment, to assess β-sheet formation (Vega et al., 2018). We noticed a significant increase in ThS signal when EFhd2 and K19 tau co-incubated in the absence of an external aggregation inducer, such as heparin. Interestingly, ThS signal remarkably diminished upon deleting the C-C domain from EFhd2 (Vega et al., 2018). Considering the liquid-liquid phase separation (LLPS) phenomenon of tau, we tested whether EFhd2 could impact tau’s demixing capabilities. Our previous findings evinced EFhd2 transforms the dynamic tau liquid droplets into static solid-like structures (Vega et al., 2019). Again, EFhd2’s C-C domain mediated the observed changes on tau’s dynamics. Recently, we reported the unique capacity of EFhd2 to interact with and entangle *in vitro*-formed tau filaments into distinct large aggregates (Soliman et al., 2024). This phenomenon has not been reported with other tau-interacting proteins.

Based on our previous research, we hypothesize EFhd2 contributes to the modulation of tau aggregation states *in vivo*. To investigate this hypothesis, we generated the Tau_*P301L*_/ that *Efhd2*^–/–^ mouse model by crossing JNPL3 mice, which express the human P301L tau mutation, with *Efhd2*^–/–^ mice. In this model, the absence of EFhd2 produced only modest changes in the age-related behavioral phenotype associated with Tau_*P301L*_ expression. However, we observed region-dependent shifts in tau pathology. In aged mice, *Efhd2* deletion was associated with reduced levels of late-stage tau markers (pSer422 and PHF1) in the cortex, whereas early conformational tau (Alz50-positive) showed higher accumulation in both the brainstem and cortex. Together, these findings suggest that the absence of EFhd2 favors the buildup of pretangle tau species while diminishing markers of more advanced tau aggregation. This pattern highlights a potential role for EFhd2 in shaping the progression of tau pathology, warranting further investigation into whether its influence on tau aggregate maturation contributes directly or indirectly to tau-mediated neurodegeneration. The Tau_*P301L*_/*Efhd2*^–/–^ mouse model provides a valuable platform for dissecting the mechanistic contribution of EFhd2 to these processes.

## Materials and methods

### Animals

**FIGURE 1:**
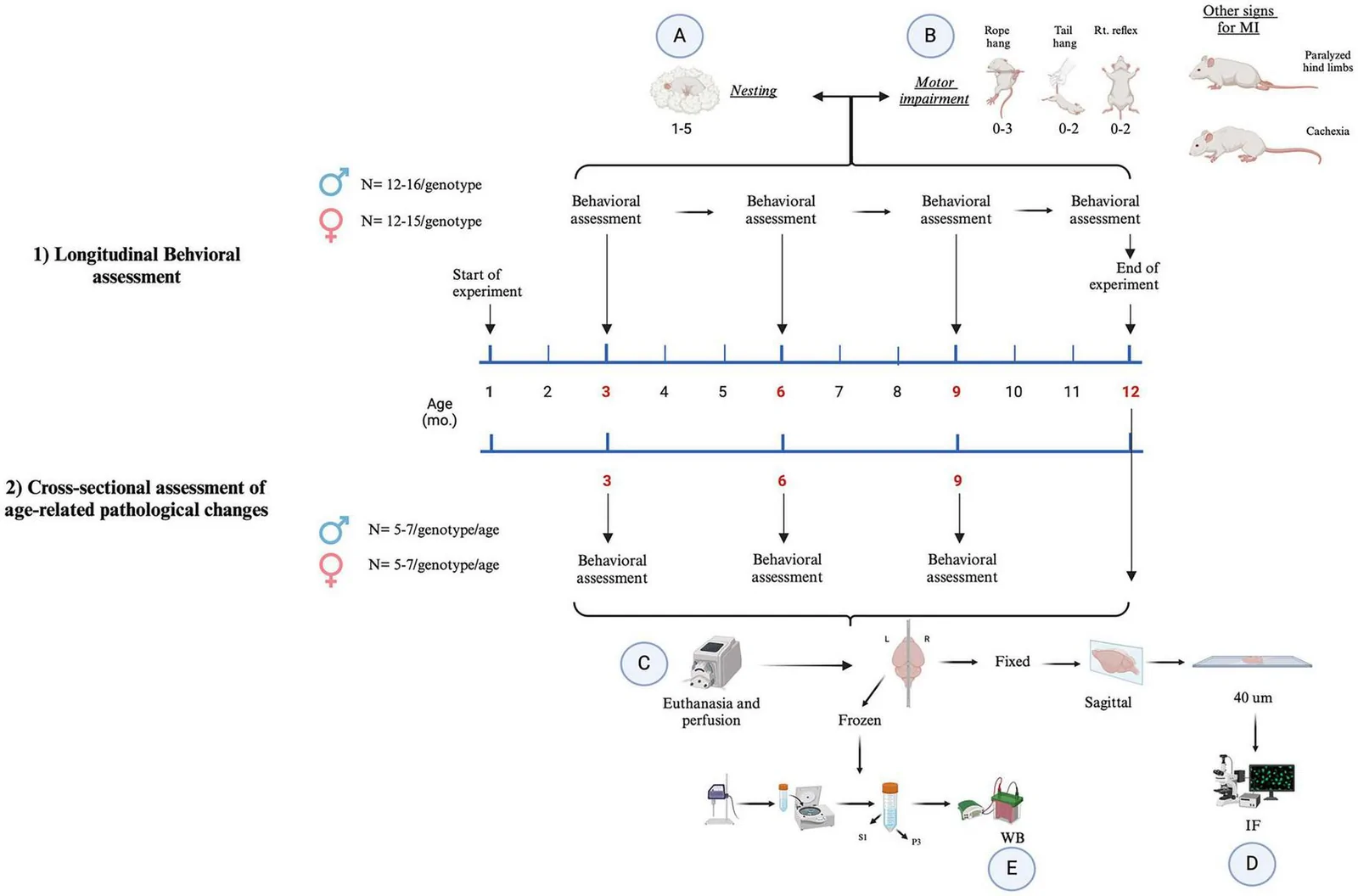
Experimental design and workflow. To investigate the impact of deleting *Efhd2* on tau pathology, we designed a two-pronged study: (1) longitudinal behavioral assessment, (2) cross-sectional assessment of age-related pathological changes. For the longitudinal behavioral assessment, we used four genotypes Non-Tg/*Efhd2^+/+^*, Non-Tg/*Efhd2*^–/–^, Tau_P301L_/*Efhd2^+/+^*, and Tau_P301L_/*Efhd2*^–/–^ males and females. Sample size was calculated according to *a priori* power analysis *n* = 12–16 per group. Behavioral assessment was conducted every 3 months starting at 3 months of age until 12 months. The behavioral tests included **(A)** Nesting behavior and **(B)** motor impairment. Other signs of motor dysfunction were noted, such as hind limbs paralysis and cachexia, which indicate severe illness and impending death. Mice were euthanized after behavioral assessment at 12 months in (2) cross-sectional study, females and males from the four genotypes with *n* = 5–7/group were assessed for behavior and euthanized immediately afterwards at 3, 6, 9, and 12 months. In **(C)** mice were euthanized and brains were collected. The right hemisphere was fixed for **(D)** immunofluorescence processing, and the left hemisphere was frozen for further homogenization for biochemical analysis by western blot **(E)**. The Figure was created with Biorender.com.

All experiments were conducted in compliance with federal, state, and institutional guidelines and approved by the Michigan State University Institutional Animal Care and Use Committee (Protocol #PROTO202400017). *Efhd2*^–/–^ mice were generated as delineated in (Soliman et al., 2021). JNPL3 mice (Tau_*P301L*_) overexpressing human 0N4R tau that harbors P301L mutation under mouse prion promoter (Tg(Prnp-MAPT*P301L) (RRID:MGI:6438659) were maintained in a heterozygous state and backcrossed to Swiss Webster (SWR) inbred strain (RRID:MGI:2160840RRID:MGI:2160840) (Lewis et al., 2000). JNPL3 mice were bred with *Efhd2*^–/–^ mice producing pups with heterozygous endogenous mouse *Efhd2* allele (*Efhd2*^+/–^) and either transgenic (Tau_*P301L*_) or non-transgenic (Non-Tg). Pups from F1 generation were then bred to generate littermates Non-Tg/*Efhd2*^+/+^, Non-Tg/*Efhd2*^–/–^, Tau_*P301L*_/*Efhd2*^+/+^, and Tau_*P301L*_/*Efhd2*^–/–^ that were then used in this study as illustrated in Figure 1. In this study, neither Non-Tg/*Efhd2*^+/–^ nor Tau_*P301L*_/*Efhd2*^+/–^ was analyzed. Since the progeny have mixed backgrounds (SWR and C57BL/6), a specific breeding strategy was then followed throughout generations to produce the required sample size of mice with identical homogenous background. To assess genomic background, we used the genomic marker developed by DartMouse*™*. This approach utilizes single-nucleotide polymorphisms (SNP) spread throughout the genome to determine the contribution of genomic DNA when different mouse strains are crossed, providing greater efficiency toward reaching a homogenous genomic background colony. Based on genomic marker data, the mouse colony shows a stable mixed SWR/C57BL/6 genomic background (Supplementary Figure 1A). The background was assessed every few generations to ensure background homogeneity. In that way, confounding effects due to a predominant background that could impact data interpretation was avoided. Mice were socially housed (up to 5 mice per cage) without enrichments in a 12-h light/dark cycle with food and water provided ad libitum. Mice were transferred to a clean cage with food and water weekly.

#### Genotyping

DNA was extracted from ear punches at weaning (21 days) using Kappa Mouse Genotyping Kit (GE cat. no. KK7352) according to manufacturers’ recommendations. Amplification of the LacZ gene was performed using the 3’ Uni *Neo* (*5’****-****GCAGCCTCTGTTCCACATACACTTCA-3’*) and Reg 10032R1 (*5’-GCCTATAGTTAAGGGGAGTTGGGTGG-3’*) primers. For the *Efhd2* gene, Fwd (*5’****-****CTTGGCCTCGAAGAAGTTCTTGG-3’*) and Rev (*5’****-****GCCCTCTAAGGCTTTGTGAATGC-3’*) primers were used. For *Mapt* gene, Exon 9 Fwd (*5’-CACTGAGAACCTGAAGIACCAG-3’*) and Exon 13 Rev (*5’-AGACACCACTGGCGACTTGTAC-3’*) primers were used. Amplification of the three genes was performed using cycling conditions recommended by the KOMP consortium. The PCR reaction constitutes 12.5 μl 2x Kappa Fast genotyping, 1.25 μl Primer Fwd (100 ng/μl), 1.25 μl primer Rev (100 ng/μl), 1 μl extracted DNA and 9 μl ddH_2_0. The amplified DNA was then separated on 1% agarose and visualized with 0.005% ethidium bromide using Bio-Rad Imager as shown in Supplementary Figure 1B. Positive control represents a verified *Tau_*P301L*_/*Efhd2*^+/–^* whereas the negative control is the master mix (including the primers) without DNA sample (Supplementary Figure 1B).

#### Sample size

*A priori* power analysis was conducted using G*Power (RRID:SCR_013726) (Faul et al., 2007; Kang, 2021). First, preliminary behavioral data were used to calculate effect size with PASS 2020 Power Analysis and Sample size Calculation (RRID:SCR_019099). Due to sample size limitation, the power analysis did not include sex as a factor. Hence, no statistical comparison between males and females was conducted in this study. The calculated sample size for longitudinal behavioral assessment using effect size 0.44 for repeated measures ANOVA (RM-ANOVA), 80% power and α 0.05 was 11 mice/genotype/sex. Given the potential for attrition and to ensure adequate power, we aimed for 16 mice/genotype/sex as depicted in Figure 1. By the end of the study, the sample size was 12–16/group. The sample size for the cross-sectional study (Figure 1) was estimated according to previous experiments and published research (Apicco et al., 2018). According to effect size of 0.4 for a two-way ANOVA, 80% power, and α 0.05, the calculated sample size was 4/genotype/age/sex. We aimed for 6/group to compensate for untoward technical issues (Figure 1). Collectively, the sample size determined for the entire study was approximately 320 mice.

It is important to note that randomization was not possible in this study; however, experimenters were blind to the genotype of each mouse during behavioral and pathological assessment. After data collection, genotypes were decoded.

#### Behavioral testing

In the longitudinal section, mice were aged to 12 months, whereby behavioral assessment was undertaken every 3 months starting at the age of 3 months. In the cross-sectional behavioral and pathological analysis, separate cohorts of mice were aged at 3, 6, 9, and 12 months. Immediately after the behavioral assessment of each age, mice were euthanized, and tissues were collected as shown in Figure 1.

*1. Motor function assessment*

Motor phenotype was represented by motor impairment score (MIS) that was assessed according to three tests: tail hang (scale 0–2), righting reflex (score 0–2), and rope hand (score 0–2) (Lewis and McGowan, 2005). The rope hang test was performed twice. Each score was given according to a rubric established in (Lewis and McGowan, 2005). The scores of the three tests were combined and converted to a final MIS, which equates to the respective stage of neurodegeneration (Lewis and McGowan, 2005). Each mouse was evaluated by two independent raters who were blinded to the genotype, and the average of their scores was used for analysis. The assessment of motor function was conducted after the nesting test. In addition to MIS, other signs of motor dysfunction were monitored weekly starting at 6 months, including slouched posture, conjunctivitis, dystonia, paralyzed hind limbs, and cachexia. These signs typically indicate severe illness followed by death. The mice that died before the end of the study (12 months) due to motor phenotype were given score 30 of MIS and score 1 for nesting and are calculated as death events for survival analysis.

*2. Nesting*

In this study we assessed nesting behavior to evaluate impaired activities of daily living that reflect cognitive function (Deacon, 2006; Neely et al., 2019). All mice for longitudinal and cross-sectional experiments were evaluated for nesting performance. Evaluating nesting performance was adopted from a previously described protocol (Deacon, 2006). Briefly, mice were transferred to a clean cage individually with no enrichment except standard Neslets 5 cm^2^ cotton batting 24 h before the test. On day 2, nesting construction was evaluated according to the 5-point scale described in (Deacon, 2006). Each mouse was assessed by three independent blind raters; then, the average score was used for subsequent analysis.

#### Combined phenotype score

We sought to create a combined score that represents nesting and motor phenotype for each mouse. This combined score better represents the partial dependence of nesting behavior on motor function that we observed in aged mice. Although nesting construction does not require a high level of motor coordination, in contrast to mazes, terminally ill mice show markedly impaired motor function that affects nesting construction. More importantly, the rarity of some raw MIS within groups hindered the analysis by ordinal logistic regression. Hence, the combined score circumvented this problem by transforming the scores into continuous data that could be analyzed using mixed-factor ANOVA. To this end, we first normalized each of nesting score (NS) and MIS using conventional, minimum-maximum normalization methods as follows:


$$\mathrm{Normalized}\,\mathrm{MIS}=\frac{30-\mathrm{score}}{30}$$

$$\mathrm{Normalized}\,\mathrm{NS}=\frac{\mathrm{score}-1}{4}$$

Then we combined them in an equation to calculate the final phenotype score:


C⁢o⁢m⁢b⁢i⁢n⁢e⁢d⁢p⁢h⁢e⁢n⁢o⁢t⁢y⁢p⁢e⁢s⁢c⁢o⁢r⁢e=(N⁢o⁢r⁢m⁢a⁢l⁢i⁢z⁢e⁢d⁢M⁢I⁢S*0.5)+

(N⁢o⁢r⁢m⁢a⁢l⁢i⁢z⁢e⁢d⁢N⁢S*0.5)

In this equation, 0.5 denotes the weight that each of NS and MIS contributes to the final phenotype. The combined phenotype score could reside between 0 and 1 where 0 indicates severe illness and behavioral phenotype, whereas 1 indicates normal phenotype. Analyzing the data as combined phenotypes score does not obscure the conclusions when juxtaposed with the raw scores (Supplementary Figure 2).

#### Tissue collection and processing

At the age of sacrifice, mice were administered intraperitoneally an overdose of Fatal-Plus solution (pentobarbital sodium) (≥100 mg/kg). Mice were transcardially perfused with 0.9% saline containing heparin (10,000 units/L) at rate 2 ml/min for 20 min. Brains were then removed and the right hemisphere was post-fixed in 4% paraformaldehyde overnight at 4 °C. For cryoprotection, brains were then immersed first in a 15% sucrose solution for 24 h (until sinking to the bottom of the vial), followed by a 30% sucrose solution. The left hemisphere was removed, and flash frozen on dry ice for downstream biochemical analysis. Using a freezing stage and sliding knife microtome, the right hemisphere was sliced into 40 μm sagittal sections. Brain sections were stored in a cryoprotectant solution at 4 °C (30% sucrose + 30% ethylene glycol in PBS) for subsequent histological analysis.

### Immunofluorescence

Initial tittering experiments were conducted to determine optimal primary antibodies dilution. DAPI, a nuclear marker (Thermo Scientific cat. no. D1306) was stored as a stock solution of 5 mg/ml in DMSO (Sigma cat. no. D8418) and frozen at −20°C. Immunofluorescence was undertaken according to established protocols (Combs et al., 2016). The 40-μm free floating tissues were washed in 0.1%Triton-X (Thermo Scientific cat. no. A16046) in TBS for 6 times x 10 min. Then, tissues were blocked in 10% goat serum/2% BSA/0.4% Triton-X/TBS for 1.5 h at room temperature (goat serum: Gibco cat. no. 16210-072; BSA: Bioreagents cat. no. BP1600). Primary antibodies were diluted in 2% goat serum/0.1% Triton-X/TBS: pSer422 (1:2K; Abcam cat. no. ab79415, RRID:AB_1603345), Alz50 (1:2K, RRID:AB_2313937), and PHF1(1:500, RRID:AB_2315150) (Wolozin et al., 1986; Ksiezak-Reding et al., 1988, 1995; Goedert et al., 1991; Greenberg et al., 1992; Otvos et al., 1994; Carmel et al., 1996). After blocking, tissues were incubated with the primary antibody overnight at 4°C. Then the tissues were washed in 0.1% Triton-X/TBS 6 times × 10 min. Afterwards, tissues were incubated with secondary antibody 1:500: AlexaFluor goat anti-mouse IgG1 (H + L) 488 (Invitrogen, cat. no. A-21121, RRID:AB_2535764), AlexaFluor goat anti-mouse IgM (H + L) 488 (Invitrogen, cat. no. A-21042, RRID:AB 2535711), and AlexaFluor goat anti-rabbit IgG (H + L) 488 (Invitrogen, cat. no. A-11008, RRID:AB_143165) for 2 h at room temperature. The first wash after secondary antibody contained DAPI at a final concentration of 0.5 ng/μl in 0.1% Triton-X/TBS followed by 5 washes × 10 min. Sections were then mounted on microscope slides and allowed to air dry overnight. To block autofluorescence, mounted sections were stained with Sudan Black (Thermo Scientific, cat. no. BP109). Briefly, slides were rinsed in water by 3 dips followed by equilibration in 70% ethanol for 2 min. Then the slides were covered with 2% Sudan Black/70% ethanol for 5 min. Immediately afterwards, the slides were rinsed with 3 dips of 70% ethanol followed by 3 quick dips in water. Finally, slides were washed in water twice x 1 min. Slides were quickly coverslipped with Vectashield aqueous mounting media (Vector laboratories, cat. no. H-1000). All slides were stored in the dark at 4 °C until ready for imaging.

Immunofluorescence (IF) images were acquired with ZEISS Axio Scan.Z1 Slide Scanner (RRID:SCR_020927) using the appropriate fluorescence filter for each fluorophore. Area of positive stain was quantified using Halo (Indica Labs) (RRID:SCR_018350), Area Quantification FL v2.3.3 module. Using Allen Mouse Brain Reference Atlas (RRID:SCR_002978), three sections per animal were selected encompassing the brainstem, hippocampus, and cortex. These three regions were quantified separately for positive stain area. Negative controls Non-Tg brain sections were used to determine the background and ensure antibodies specificity.

### Western blotting

Left brain hemispheres were weighed and homogenized in 5 volumes (w/v) ice-cold Buffer A (25 mM Tris–HCl, pH7.4/150 mM NaCl/1 mM EDTA/1 mM EGTA/5 mM sodium pyrophosphate/30 mM β-glycerophosphate/30 mM sodium fluoride/1 mM phenylmethylsulfonyl fluoride (PMSF) with 1X Halt protease inhibitors cocktail (Thermo Scientific, cat. no. 78430). Subsequently, the homogenates were centrifuged using Sorvall MTX 150 Micro-Ultracentrifuge at 110,000×*g* (rotor S120-AT2) at 4 °C for 15 min. The supernatant (S1) fraction was carefully transferred to clean tubes. Pellets (P1) were resuspended in 5 volumes (w/v) ice-cold Buffer B (20 mM Tris-Base pH 7.4/800 mM NaCl/1 mM EDTA/1 mM EGTA/1 mM PMSF/5 mM sodium pyrophosphate/10 mM β-glycerophosphate/30 mM sodium fluoride/10% sucrose) and sonicated using Misonix XL-2000 set at 4 on ice 3 times with 3 s pulses. Homogenates were then ultracentrifuged at 110,000×*g* at 4 °C for 15 min. With great caution, the supernatants (S2) were transferred to clean tubes. Sarkosyl (Acros Organics cat. no. 61207-5000) (10% w/v) was added to the S2 fractions to a final concentration of 1%, vortexed, and incubated at 37 °C for 1 h. Then S2 fraction was ultracentrifuged at 110,000 x g for 30 min at 4 °C. Supernatants (S3) were carefully removed and stored at −80 °C. The pellets (P3, sarkosyl-insoluble fraction) were resuspended in 50 μl Buffer A. Protein concentration of S1 fraction was determined using Pierce BCA Protein Assay Kit (cat. no. 23225). To avoid repetitive freeze-thaw cycles, separate aliquots of S1 and P3 were prepared and stored at −80 °C until immunoblotting.

For all P3 samples, 3 μl were mixed with 2X SDS loading buffer. The amount of protein loading for S1 samples varied according to each primary antibody ranging from 1.5 to 10 μg protein mixed 2X SDS loading buffer. Protein was separated on 4–15% Criterion TGX Stain-Free gels (Bio-Rad cat. no. 5678084) and transferred to pure nitrocellulose membrane (0.45 μm Bio-Rad cat. no. 1620115). The membrane was blocked using 5% dry-milk solution in 1x TBST (2.5 mM Tris-Base/15 mM NaCl/30 mM KCL/0.02% Tween 20 detergent) for 1h at room temperature. Then the membrane was incubated with the primary antibodies overnight at 4 °C. The primary antibodies used are mouse monoclonal anti-Tau13 (1:100k, Biolegend, cat. no. 835201, RRID:AB_2565341), mouse monoclonal anti-PHF1 (1:1000, RRID:AB_2315150), mouse monoclonal anti-EFhd2, clone 10D6 (1:5000, prepared by Kanaan lab (Soliman et al., 2021)), and rabbit monoclonal pSer422 (1:1000, Abcam cat. no. Ab79415, RRID:AB_1603345). The next day, the membrane is washed 3 times × 5 min in 1x TBS-T before incubation with secondary antibodies: goat anti-rabbit IRDye 680 (LI-COR cat. no. 926–68021, RRID:AB_10706309) and goat anti-mouse IRDye 800 (LI-COR cat. no. 926–32210, RRID:AB_621842) at 1:2000. Afterwards, the membrane is washed 3 times x 5 min in TBS-T and imaged by LI-COR Odyssey Classic Imager (RRID:SCR_023765), using LI-COR Image Studio Software (RRID:SCR_015795) (v5.2).

### Statistical analysis

All data were analyzed using GraphPad Prism v10.2.2 (San Diego, www.graphpad.com, RRID:SCR_002798). Repeated-measures mixed-factor ANOVA (RM-ANOVA) was used to evaluate group differences in longitudinal behavioral measures. Tukey’s post hoc tests were applied for pairwise comparisons. For cross-sectional histological and biochemical data, group differences were analyzed using two-way ANOVA followed by Holm–Šídák post hoc tests.

For all RM-ANOVA and two-way ANOVA models, partial eta squared (η^2^*p*) values were calculated to quantify the magnitude of main effects and interactions (Maher et al., 2013). Effect sizes were interpreted using Cohen’s (2013) guidelines: negligible (<0.01), small (0.01), medium (0.06), and large (0.14). When comparing specific groups following post hoc tests, Hedges’ g was calculated and interpreted as small (0.2), medium (0.5), or large (0.8) (Lakens, 2013; Maher et al., 2013). All analyses used a 95% confidence interval. Significance thresholds were *p < 0.05, **p < 0.01, ***p < 0.001, and ****p < 0.0001. Data are presented as mean ± SEM.

Survival differences were assessed using Kaplan–Meier analysis with the log-rank (Mantel–Cox) test and a family-wise α of 0.05. Pairwise survival comparisons were corrected using Bonferroni-adjusted *p*-values (α = 0.025).

## Results

### Survival analysis of Tau_P301L_/*Efhd2*^–/–^ mice

In this study, we sought to investigate the impact of EFhd2 on progressive pathology and behavioral deficit induced by tau. To this end, we bred *Efhd2^–/–^* mice with JNPL3 (Tau_P301L_) mice. The JNPL3 transgenic mouse model overexpresses 0R4N human tau that harbors P301L mutation under the control of mouse prion promoter (Lewis et al., 2000). Pathological tau accumulation in the form of neurofibrillary tangles (NFTs) and pretangles in addition to neuronal loss predominate primarily in spinal cord, brainstem, and telencephalon (Lewis et al., 2000; Lewis and McGowan, 2005). Therefore, motor impairment is the principal pathological phenotype in this Tau_P301L_ expressing mouse model. That motor phenotype indeed overlaps with motor symptoms in a few tauopathies as PSP, FTDP, and ALS (Lewis and McGowan, 2005). Several studies have established the sex difference in mice expressing Tau_P301L_ where females develop tau pathology and exhibit the impaired motor phenotype approximately 6 months earlier than males (Sahara et al., 2002; Lewis and McGowan, 2005). Moreover, the severity of motor impairment corresponds to the neuronal burden of tau aggregates and escalates with advanced aging.

Shorter lifespan due to severe motor impairment characterizes Tau_P301L_ mice. Particularly, the mice become moribund within 4-8 weeks from initial signs of motor impairment (Lewis et al., 2000). In this study, we conducted Kaplan-Meier survival analysis to examine whether the absence of EFhd2 changed the lifespan of Tau_P301L_ expressing mice. In addition to collecting NS and MIS every 3 months, mice were monitored for other motor impairment signs, including dystonia, hunched posture, conjunctivitis, and cachexia. The progression of these signs ultimately hindered the mice from properly reaching water and food, leading to death.

**FIGURE 2:**
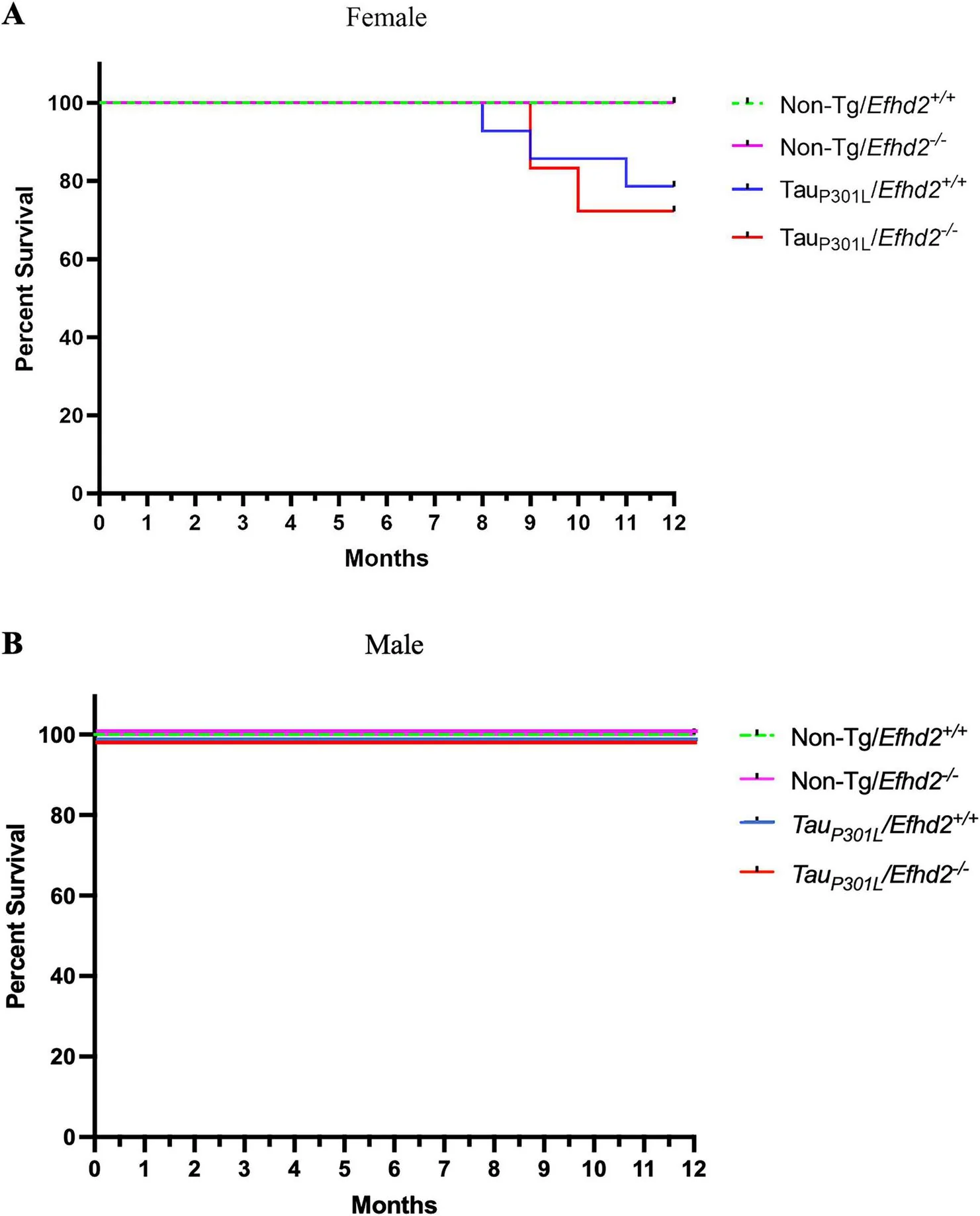
Deleting *Efhd2* gene did not change the lifespan of Tau_P301L_ mice. **(A)** Kaplan–Meier survival curve of females indicated the difference in percent survival among groups by the log-rank (Mantel–Cox test) (*p* = 0.0431). Pairwise comparison revealed a reduction in percent survival of Tau_P301L_/*Efhd2^+/+^* mice compared to Non-Tg/*Efhd2^+/+^* mice (*p* = 0.0719). Likewise, comparing the survival curve of Tau_P301L_/*Efhd2^+/+^* and Tau_P301L_/*Efhd2*^–/–^ mice by log-rank (Mantel–Cox test) detected a difference (*p* = 0.7219). At 12 months, the percent survival of Tau_P301L_/*Efhd2*^–/–^ mice was 72.2% vs. 78.5 % in Tau_P301L_/*Efhd2^+/+^* mice. Non-Tg mice did not face any death events until the end of the study, indicating the deleting *Efhd2* gene did not provoke early death. Non-Tg/*Efhd2^+/+^ n* = 14, Non-Tg/*Efhd2*^–/–^ *n* = 14, Tau_P301L_/*Efhd2^+/+^ n* = 14, Tau_P301L_/*Efhd2*^–/–^ *n* = 18. **(B)** Visualization of lifespan of male mice. Herein, Kaplan–Meier survival curve analysis of males was not feasible due to the absence of death events. Until the end of the study at 12 months no neurodegeneration-related death event was recorded.

Kaplan–Meier curves are shown in Figure 2 for females and males. Among females (Figure 2A), the log-rank (Mantel–Cox) test indicated an overall difference across survival curves (*p* = 0.043). Pairwise comparisons showed (i) lower percent survival in Tau_P301L_*/Efhd2^+/+^* compared with Non-Tg/*Efhd2^+/+^* (*p* = 0.0719) and (ii) no detectable difference between Tau_*P301L*_/*Efhd2^+/+^* and Tau_*P301L*_/*Efhd2^–/–^* (*p* = 0.7219). None of the pairwise comparisons met the Bonferroni-adjusted threshold (α = 0.025 for two comparisons); hence, we could deduce an overall directional change rather than a categorical difference. Consistent with these patterns, the earliest motor-related death events occurred at ∼8 months in Tau_P301L_/*Efhd2^+/+^* females and ∼9 months in Tau_P301L_/*Efhd2^–/–^* females. By 12 months, percent survival was moderately similar between Tau_P301L_/*Efhd2^–/–^* (72.2%) and Tau_P301L_/*Efhd2^+/+^* (78.5%; *p* = 0.72). In males (Figure 2B), no motor-related death events were observed over the study’s duration. These findings accord with the previously reported delayed onset of overt phenotypes in males (12–15 months) (Lewis et al., 2001; Sahara et al., 2002; Arendash et al., 2004; Rao et al., 2014). Importantly, comparison of Non-Tg/*Efhd2^+/+^* and Non-Tg/*Efhd2^–/–^* indicated that *Efhd2* deletion alone did not measurably affect lifespan or precipitate early death events (Purohit et al., 2014). Taken together, these data point to modest, sex-specific differences in survival patterns associated with Tau_P301L_ expression, with no evidence that *Efhd2* deletion independently alters lifespan.

### Behavioral assessment of Tau_P301L_/*Efhd2*^–/–^ mice

Tau_*P301L*_ mice overexpress human 0N4R tau with P301L mutation under the control of the mouse prion promoter (Lewis et al., 2000). The original JNPL3 model was developed after the initial discovery of FTDP-17-related missense mutations (Hutton et al., 1998). The progressive pathological tau accumulation starts in the brainstem and spinal cord before later propagation to other brain regions; primarily the cortex, hypothalamus, and basal ganglia (Lewis et al., 2000; Sahara et al., 2002). In this model, less pathology is noted in the hippocampus. As a result, the principal behavioral phenotype in this model is motor impairment exhibited as progressive dystonia of hind limbs, abnormal escape extension, hunched posture, and inability to righting. This motor phenotype does not represent all human tauopathies but overlaps with progressive supranuclear palsy (PSP), FTD-ALS, and FTDP-17. In fact, the severity of motor impairment increases with advanced age in JNPL3 mice and correlates with pathological tau depositions and neurodegeneration.

Since the main behavioral phenotype in the Tau_*P301L*_ model is age-dependent motor dysfunction, traditional maze-based behavioral tests, such as water maze or Y-maze, to assess cognitive domain in this model become unreliable. Alternatively, in this study we assessed nesting behavior as a substitute to evaluate impaired activities of daily living that reflect cognitive function (Deacon, 2006; Neely et al., 2019).

**FIGURE 3:**
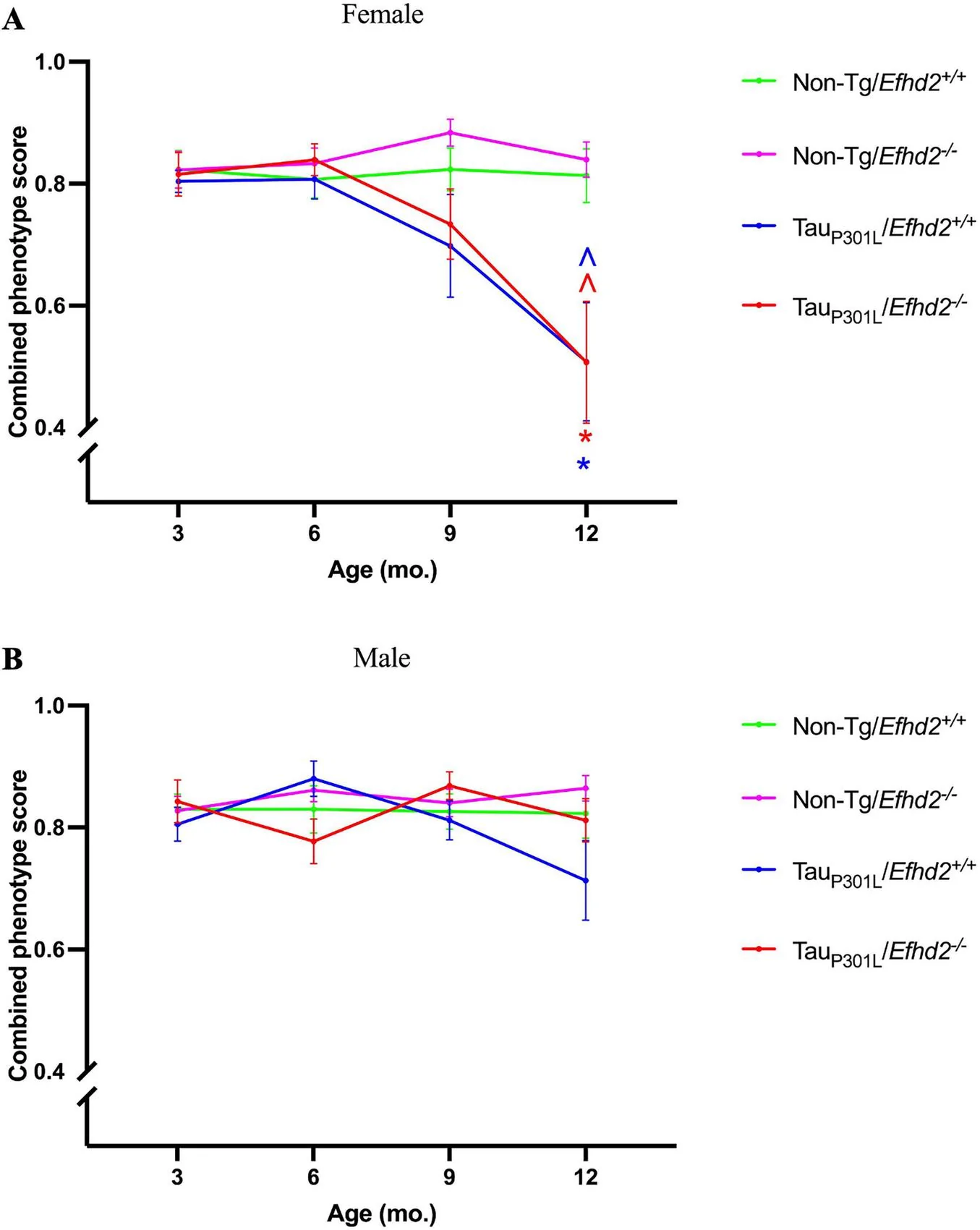
Age-dependent changes in combined behavioral phenotype in Tau_*P301L*_ mice with or without *Efhd2*. NS and MIS were normalized and combined to generate a composite phenotype score (see “Materials and methods”). **(A)** In female mice, Tau_*P301L*_ animals exhibited a clear age-dependent decline in phenotype scores, most evident at 12 months. This decline was observed in both *Efhd2*^+/+^ and *Efhd2*^–/–^ groups, with no substantial effect of *Efhd2* deletion on the overall trajectory. Non-transgenic groups showed minimal changes across time. **(B)** In male mice, phenotype scores displayed greater variability across ages, with no consistent pattern associated with *Efhd2* deletion. Data are presented as mean ± SEM (*n* = 12–16). Detailed statistical analyses and all corresponding values are reported in the “Results” section.

Longitudinal assessment of motor function and nesting was executed every 3 months starting at 3 months as described in the methods section. NS and MIS were normalized and combined with a final phenotype score that provides a holistic evaluation of phenotypic abnormalities in the mice (See “Materials and methods: combined phenotype score”). To support this approach, we included a Supplementary Figure 2 presenting the raw MIS and NS scores separately. This allows direct comparison with the combined phenotype score and confirms that the main conclusions remain consistent. It is important to note that the mice that died before the end of the study due to severe motor dysfunction were considered terminally ill and given MIS and NS representing severe disease stage. Figure 3 demonstrates data analysis for the combined pathological phenotype score using RM-ANOVA to detect the differences among groups in females and males. A large number of studies reported invariably sex differences in regard to *phenotype onset in Tau_*P301L*_*; hence, we conducted a separate analysis for males and females.

We can observe from Figure 3A an overall decline in the phenotype score in both Tau_P301L_/*Efhd2^+/+^* and Tau_P301L_/*Efhd2^–/–^* females compared to their Non-Tg littermates. Mixed-factor RM-ANOVA identified a large interaction between genotype and age [F(9, 153) = 4.649, *p* < 0.0001, η^2^*p* = 0.21], indicating that changes in phenotype scores over time differed across genotypes. The analysis also detected large main effects of age [F(2.118, 108.0) = 12.80, *p* < 0.0001, η^2^*p* = 0.20] and genotype [F(3, 51) = 3.937, *p* = 0.0133, η^2^*p* = 0.18].

By isolating simple main effects, age exerted large effects on phenotype scores in both Tau_P301L_/*Efhd2*^+/+^ [F(1.595, 17.54) = 6.854, *p* = 0.0092, η^2^*p* = 0.38] and Tau_P301L_/*Efhd2*^–/–^ female mice [F(1.707, 22.19) = 9.527, *p* = 0.0016, η^2^*p* = 0.42]. In Non-Tg mice, age had a negligible effect in Non-Tg/*Efhd2*^+/+^ [F(2.671, 37.39) = 0.067, *p* = 0.9680, η^2^*p* < 0.01] and a medium effect in Non-Tg/*Efhd2*^–/–^ [F(2.868, 37.28) = 1.19, *p* = 0.3249, η^2^*p* = 0.08].

Post hoc Tukey comparisons showed minimal change in Tau_P301L_*/Efhd2^+/+^* phenotype scores between 3 and 6 months (*p* = 0.9996, *g* < 0.2). Larger age-related declines appeared at 12 months when compared with 3 months (*p* = 0.0361, *g* = 1.2), 6 months (*p* = 0.0388, *g* = 1.2), and 9 months (*p* = 0.023, *g* = 0.6). Tau_P301L_/*Efhd2^–/–^* mice showed a similar pattern: small change between 3 and 6 months (*p* = 0.8949, *g* = 0.2) and pronounced reductions at 12 months relative to 3 (*p* = 0.0171, *g* = 1.1), 6 (*p* = 0.0171, *g* = 1.2), and 9 months (*p* = 0.0359, *g* = 0.74).

We also analyzed the simple main effect of genotype to determine the difference in phenotype scores in each age group. Genotype differences were negligible at 3 months [F(3, 51) = 0.0869, *p* = 0.9669, η^2^*p* = 0.01] and 6 months [F(3, 51) = 0.354, *p* = 0.7861, η^2^*p* = 0.02]. A medium genotype effect emerged at 9 months (F(3, 51) = 2.582, *p* = 0.0635, η^2^*p* = 0.13), followed by a large effect at 12 months [F(3, 51) = 6.556, *p* = 0.0008, η^2^*p* = 0.28]. At 12 months, Tau_P301L_/*Efhd2^+/+^* mice showed large reductions in phenotype scores relative to Non-Tg/*Efhd2^+/+^* (*p* = 0.0233, *g* = 1.2) and Non-Tg/*Efhd2^–/–^* (*p* = 0.0135, *g* = 1.4). Tau_P301L_/*Efhd2^–/–^* female mice exhibited similarly large reductions compared to Non-Tg/*Efhd2^+/+^* (*p* = 0.0164, *g* = 1.0) and Non-Tg/*Efhd2^–/–^* (*p* = 0.0092, *g* = 1.2). No meaningful difference was observed between Tau_P301L_/*Efhd2^+/+^* and Tau_P301L_/*Efhd2^–/–^* at 12 months (p > 0.9999, g < 0.2). Overall, both Tau_P301L_ groups showed a progressive, age-dependent decline in behavioral phenotype scores, with the most pronounced reductions occurring at 12 months. Across ages, the absence of EFhd2 did not meaningfully alter the timing or trajectory of phenotype decline in Tau_P301L_ female mice.

Figure 3B illustrates the longitudinal behavioral phenotype scores for males across the four genotypes. Mixed-factor RM-ANOVA identified a medium interaction between age and genotype [F(9, 153) = 2.328, *p* = 0.0174, η^2^*p* = 0.12], indicating modest differences in how phenotype scores changed over time across groups. The main effects of genotype (F(3, 51) = 0.8907, *p* = 0.4523, η^2^*p* = 0.04) and age [F(2.519, 128.5) = 1.310, *p* = 0.2747, η^2^*p* = 0.03] were small, suggesting limited overall variation attributable to either factor alone.

When examining age effects within each genotype, Non-Tg/*Efhd2^+/+^* males showed negligible age-related change [F(2.711, 29.82) = 0.0167, *p* = 0.995, η^2^*p* < 0.01], and Non-Tg/*Efhd2^–/–^* mice showed only a small age effect [F(2.267, 38.53) = 0.7981, *p* = 0.4715, η^2^*p* = 0.04]. In contrast, both Tau_P301L_ genotypes exhibited larger age effects; Tau_P301L_/*Efhd2^+/+^* [F(1.587, 17.46) = 2.803, *p* = 0.0973, η^2^*p* = 0.20] and Tau_P301L_/*Efhd2^–/–^* [F(2.632, 31.59) = 2.311, *p* = 0.1021, η^2^*p* = 0.16]. Although the *p*-values did not meet conventional thresholds, the effect sizes indicate that age notably contributed to phenotype score variability within Tau_P301L_ males.

Visual inspection of Figure 3B indicates that these age-related effects in Tau_P301L_ mice may be driven primarily by deviations at 6 months. This interpretation is supported by simple main effects of genotype at each age: genotype differences were small at 3 months [F(3, 51) = 0.2788, *p* = 0.8404, η^2^*p* = 0.02] and 9 months [F(3, 51) = 0.7849, *p* = 0.5079, η^2^*p* = 0.04], albeit medium in magnitude at 6 months [F(3, 51) = 2.164, *p* = 0.1037, η^2^*p* = 0.11] and again at 12 months [F(3, 51) = 2.585, *p* = 0.0632, η^2^*p* = 0.13]. Post hoc comparisons confirmed this pattern: at 6 months, Tau_P301L_/*Efhd2^–/–^* males showed a large drop in phenotype scores relative to Tau_P301L_/*Efhd2^+/+^* (*p* = 0.1068, *g* = 0.87). By 12 months, the direction of this pattern reversed, with Tau_P301L_/*Efhd2^–/–^* males exhibiting moderately higher scores than Tau_P301L_/*Efhd2^+/+^* (*p* = 0.3474, *g* = 0.5).

### Assessment of pathological tau markers in Tau_P301L_/*Efhd2*^–/–^ mice

We examined neuropathological changes associated with EFhd2 deletion in Tau_*P301L*_ mice and prioritized analyses in females to align molecular readouts with the phenotype. In our cohort, females displayed a consistent, age-dependent decline in behavioral phenotype scores and modest, tau-dependent differences in survival patterns, whereas males showed a more variable longitudinal profile with later onset of overt pathology. Focusing on females therefore reduces variability related to disease timing and enhances our ability to test whether EFhd2-related shifts in behavioral trajectories correspond to changes in pathological tau. Accordingly, we quantified cortical and brainstem markers of early (Alz50) and late (pSer422, PHF1) tau pathology in female mice to evaluate whether EFhd2 deletion produces concordant molecular alterations underlying the observed age-dependent phenotype.

We conducted histological and biochemical analysis for pathological tau markers at 6 and 12 months of female Tau_P301L_/*Efhd2^+/+^* and Tau_P301L_/*Efhd2^–/–^* mice. We particularly chose these two age groups for further analysis because they could model pathological events that take place in human tauopathies. In particular, scientists and clinicians agree that pathological accumulation of tau aggregates precede clinical symptoms by years or even decades (Korczyn and Grinberg, 2024) As such, by investigating pathological tau markers at 6 months (unnoticeable neurodegenerative phenotype) and 12 months (prominent neurodegenerative phenotype), we will gain deeper insights on the influence of the absence of EFhd2 on the progressive tau accumulation.

**FIGURE 4:**
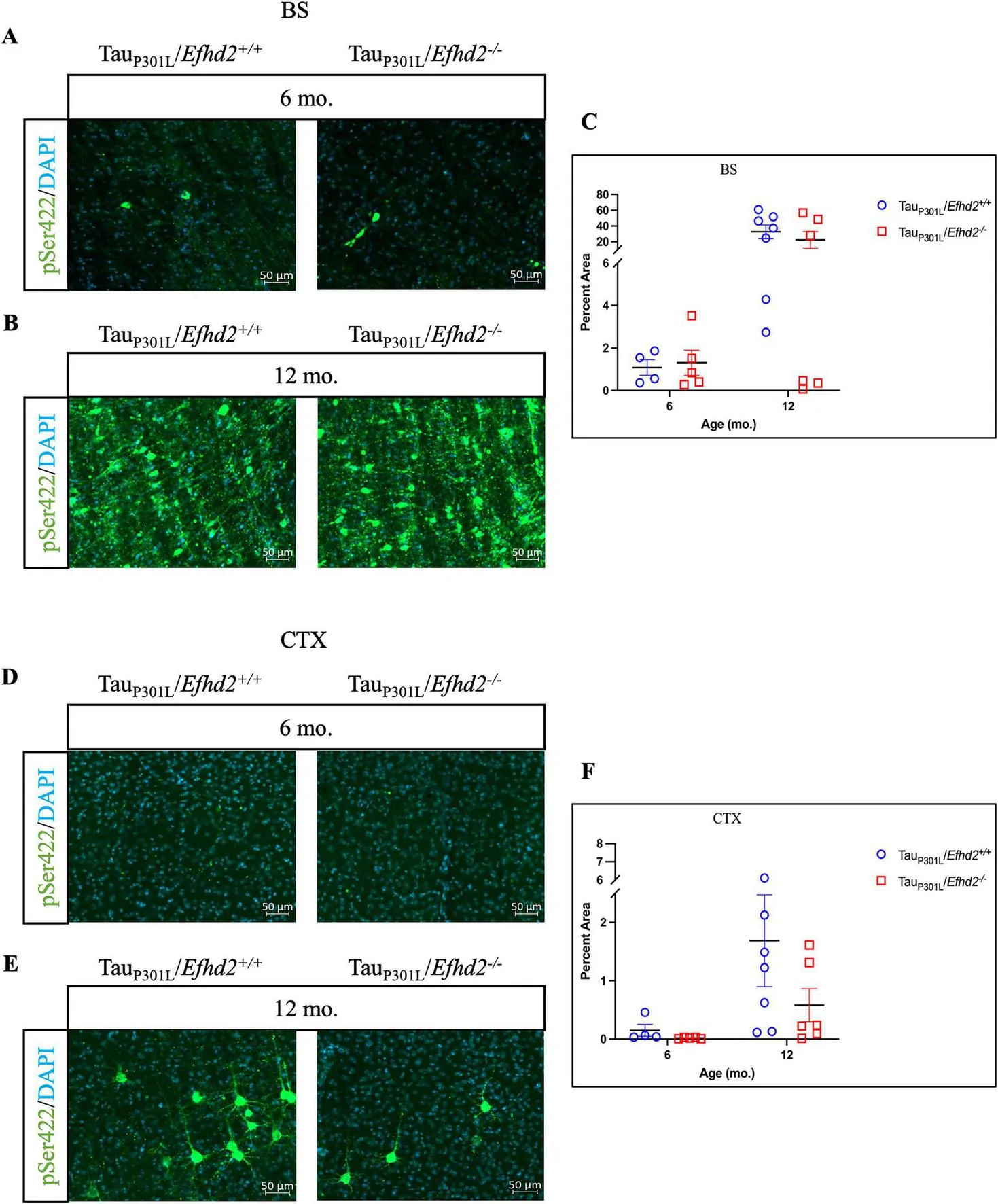
Region-specific changes in pSer422 tau staining in Tau_*P301L*_ mice with or without *Efhd2*. Right hemispheres from 6- and 12-month-old female Tau_*P301L*_/*Efhd2*^+/+^ and Tau_*P301L*_/*Efhd2*^–/–^ mice (*n* = 4–7/group) were analyzed by immunofluorescence for pSer422 tau (green) and DAPI (blue). (**A, B**) Representative images of pSer422 staining in the brainstem (BS) at 6 and 12 months. **(C)** Quantification of pSer422-positive area in the BS shows an age-dependent increase with no consistent effect of *Efhd2* deletion. (**D, E**) Representative images of pSer422 staining in the cortex (CTX) at 6 and 12 months. **(F)** Quantification of pSer422-positive area in the CTX indicates age-related changes with modest, region-specific differences between genotypes. Data are presented as mean ± SEM. Detailed statistical analyses and corresponding values are provided in the “Results” section.

First, we investigated a well-known disease-specific phosphorylated tau marker, pSer422, using IF and western blot (Figures 4 and 6). pSer422, as the name denotes, is a C-terminal phosphorylation site at serine residue 422 (Morishima-Kawashima et al., 1995). Previous studies have suggested that pSer422 modified tau hardly exists in normal brains, whereas its level remarkably increases in tauopathies brains (Hasegawa et al., 1996; Bussiere et al., 1999). Thus, pSer422 is a disease-specific pathological tau marker. Furthermore, pSer422 tau level correlates with disease progression and cognitive decline (Vana et al., 2011; Mufson et al., 2014; Kanaan et al., 2016). Immunohistochemistry-based studies on human tauopathy brains and other P301L expressing mice models substantiate pSer422 tau as a marker for intermediate and late aggregation stages (Kimura et al., 1996; Bussiere et al., 1999; Götz et al., 2001; Augustinack et al., 2002; Deters et al., 2008; Neddens et al., 2018). Strictly speaking, in the linear model of NFTs maturity, phosphorylation of tau at Ser422 reflects fibrillar and mature tangle formation with unnoticeable detection in the pretangle neurons. Typically, pretangle neurons stained by phosphorylated tau markers appear as granular or diffuse perinuclear labeling (Bancher et al., 1989; Duong et al., 1993; Braak et al., 1994).

**FIGURE 5:**
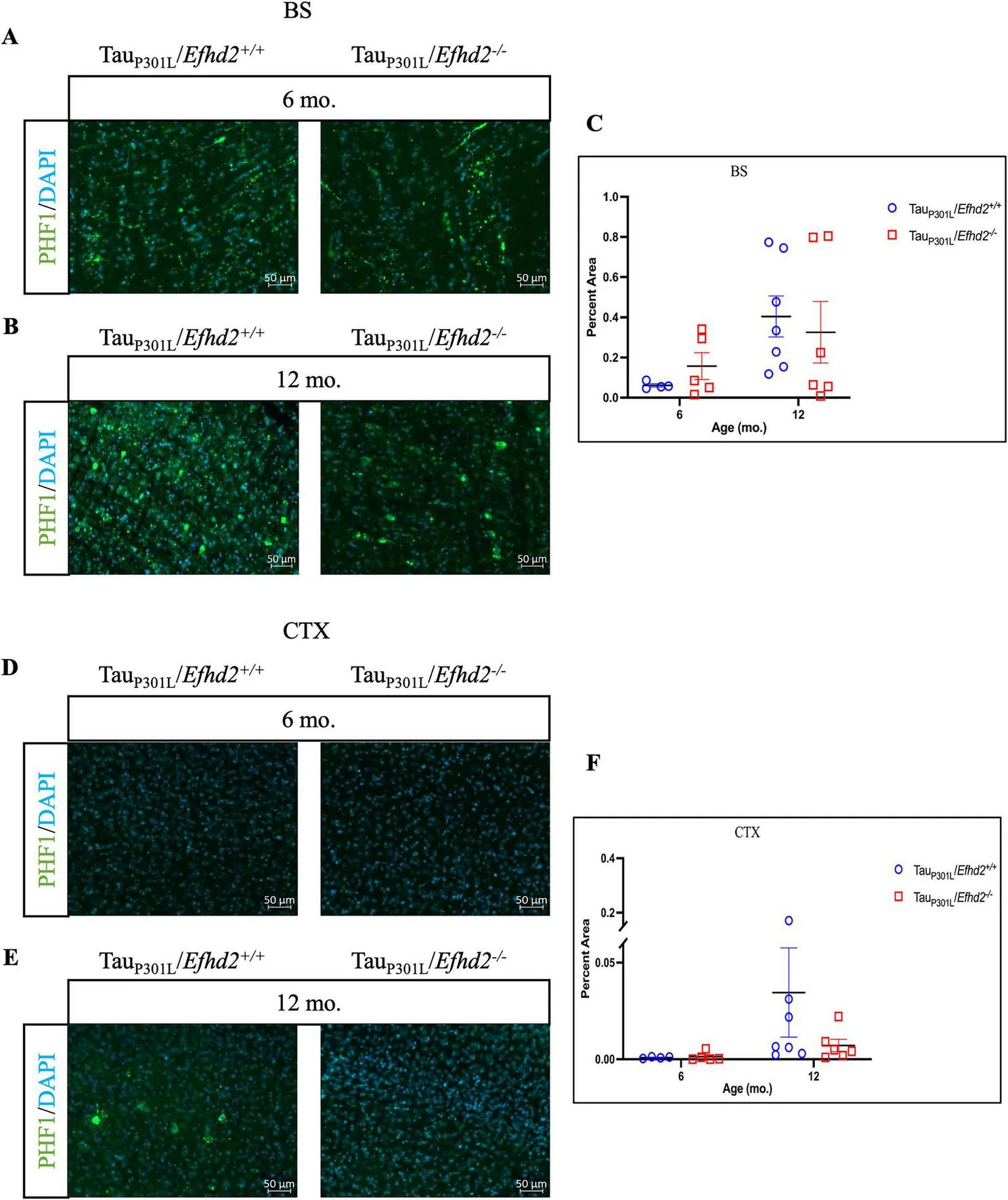
Age- and region-specific changes in PHF1 tau staining in Tau_*P301L*_ mice with or without *Efhd2*. Right hemispheres from 6- and 12-month-old female Tau_*P301L*_/*Efhd2*^+/+^ and Tau_*P301L*_/*Efhd2*^–/–^ mice (*n* = 4–7/group) were analyzed by immunofluorescence for PHF1 tau (green) and DAPI (blue). (**A, B**) Representative images of PHF1 staining in the brainstem (BS) at 6 and 12 months. **(C)** Quantification of PHF1-positive area in the BS shows an age-related increase with no clear effect of *Efhd2* deletion. (**D, E**) Representative images of PHF1 staining in the cortex (CTX) at 6 and 12 months. **(F)** Quantification of PHF1-positive area in the CTX indicates age-dependent changes, with a modest reduction in older Tau_*P301L*_/*Efhd2*^–/–^ mice compared to controls. Data are presented as mean ± SEM. Detailed statistical analyses and corresponding values are provided in the “Results” section.

**FIGURE 6:**
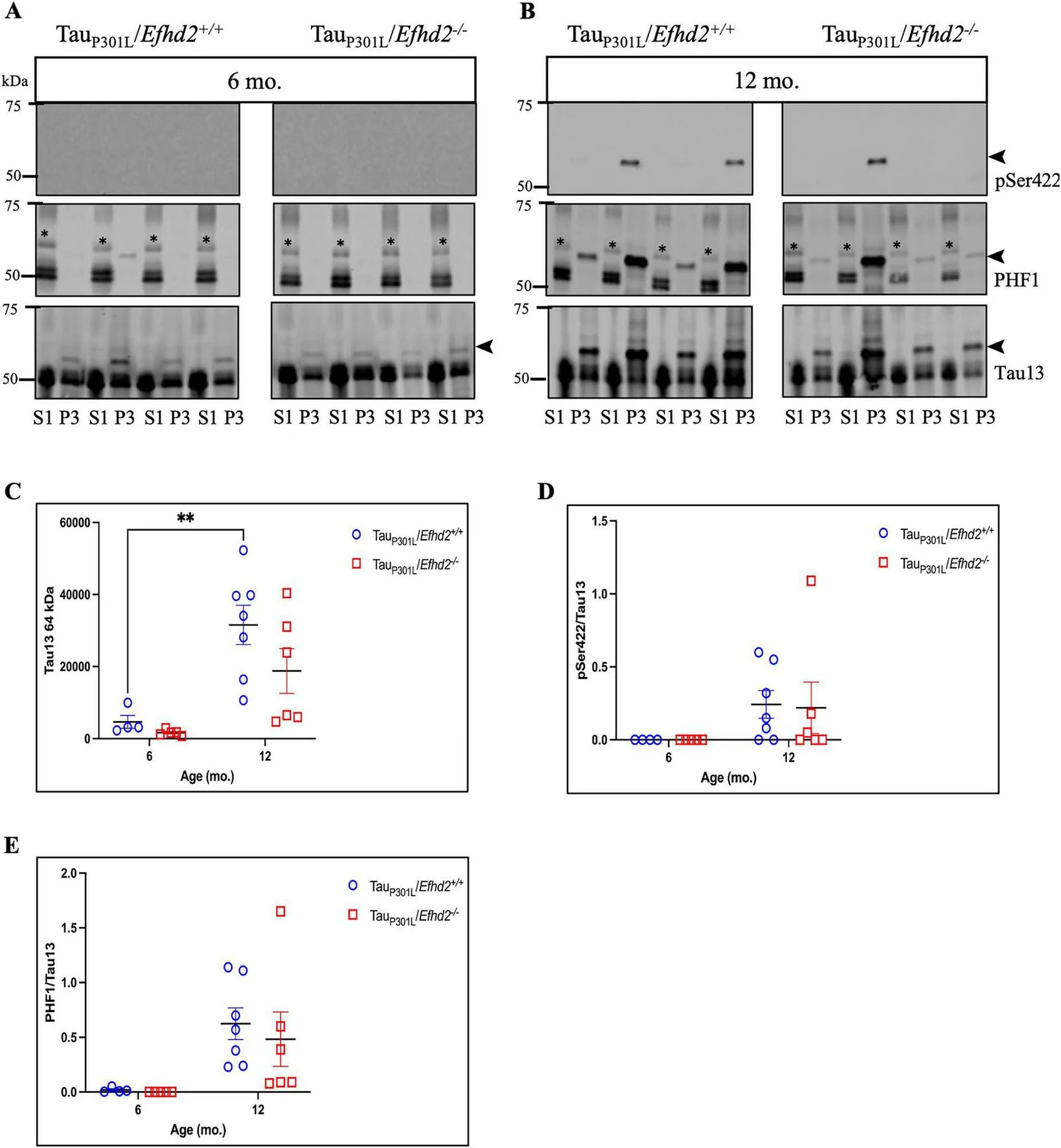
Changes in insoluble tau species in Tau_*P301L*_ mice with or without *Efhd2*. Left hemispheres from 6- and 12-month-old female Tau_*P301L*_/*Efhd2*^+/+^ and Tau_*P301L*_/*Efhd2*^–/–^ mice (*n* = 4–7/group) were fractionated into soluble (S1) and sarkosyl-insoluble (P3) fractions and analyzed by western blot. (**A, B**) Representative immunoblots for pSer422, PHF1, and Tau13 at 6 and 12 months. The arrowhead indicates the 64 kDa insoluble tau species. **(C)** Quantification of Tau13 in the P3 fraction shows an age-related increase in insoluble tau, with a reduction in Tau_*P301L*_/*Efhd2*^–/–^ mice compared to controls, particularly at 12 months. (**D, E**) Ratios of pSer422/Tau13 and PHF1/Tau13 in the P3 fraction indicate age-dependent changes without consistent effects of *Efhd2* deletion. Data are presented as mean ± SEM. Detailed statistical analyses and corresponding values are provided in the “Results” section. Immunoblots were cropped.

We confirmed that the deletion of the *Efhd2* gene did not change the selective vulnerability of brain regions as seen in Supplementary Figure 3. By comparing Tau_P301L_/*Efhd2^+/+^* and Tau_P301L_/*Efhd2^–/–^* mice, we observed no differences in the pervasion of tau depositions in different brain regions (Lewis et al., 2000). The absence of differences in brain region vulnerability was also affirmed with other pathological tau markers tested in this study.

Then, to analyze differences in stained areas, we quantified brainstem (BS) and cortex (CTX). Figure 4 represents fluorescent staining of pSer422 tau in 6 and 12 months Tau_P301L_/*Efhd2^+/+^* and Tau_P301L_/*Efhd2^–/–^* mice in BS and CTX. We observed minimal pSer422 tau reactivity in BS of 6 months mice with imperceptible difference between Tau_P301L_/*Efhd2^–/–^* and Tau_P301L_/*Efhd2^+/+^* tissues (Figure 4A). pSer422 appears as sparse staining in this age group. In contrast, pervasive pSer422 staining nearly covers the entire BS region of 12 months Tau_P301L_/*Efhd2^+/+^* and Tau_P301L_/*Efhd2^–/–^* mice as presented in Figure 4B. To assess group differences in pSer422 immunoreactivity, percent stained area was quantified and analyzed using two-way ANOVA (Figure 4C). The analysis identified a small interaction between age and genotype [F(1, 18) = 0.4025, *p* = 0.5338, η^2^*p* = 0.02], indicating that the age-related pattern of pSer422 staining was largely similar across genotypes. A large main effect of age was detected [F(1, 18) = 10.06, *p* = 0.0053, η^2^*p* = 0.36], reflecting overall increases in pSer422 staining from 6 to 12 months. In contrast, the main effect of genotype was small (F(1, 18) = 0.3682, *p* = 0.5515, η^2^*p* = 0.02).

Holm–Šídák post hoc comparisons showed a large age-related increase in pSer422 staining in Tau_*P301L*_/*Efhd2^+/+^* mice between 6 and 12 months (*p* = 0.0641, *g* = 1.7), and a similarly large increase in Tau_*P301L*_/*Efhd2^–/–^* mice (*p* = 0.2327, *g* = 1.1). Comparisons between genotypes at each age indicated only small differences: at 6 months (*p* = 0.9859, *g* = 0.2) and at 12 months (*p* = 0.3438, *g* = 0.4). Taken together, these findings indicate that pSer422 reactivity increases with age in Tau_*P301L*_ mice, but the absence of EFhd2 does not meaningfully alter the extent of pSer422 staining at either time point.

In the cortex (CTX), pSer422 staining exhibited a pattern distinct from that observed in the brainstem. At 6 months, pSer422 immunoreactivity appeared primarily as small puncta (Figure 4D), whereas by 12 months, staining was more prominent within neuronal somata and processes (Figure 4E). Quantification of percent stained area was analyzed using two-way ANOVA (Figure 4F). The analysis indicated a small interaction between age and genotype [F(1, 18) = 0.7881, *p* = 0.3864, η^2^*p* = 0.04], suggesting that age-related changes were generally similar across genotypes. A large main effect of age was detected [F(1, 18) = 3.673, *p* = 0.0713, η^2^*p* = 0.17], consistent with an overall increase in pSer422 staining from 6 to 12 months. The main effect of genotype was medium in magnitude [F(1, 18) = 1.270, *p* = 0.2746, η^2^*p* = 0.07], indicating modest differences between Tau_*P301L*_/*Efhd2^+/+^* and Tau_*P301L*_/*Efhd2^–/–^* mice.

Post hoc analyses revealed large age-related increases in pSer422 staining within each genotype: Tau_*P301L*_/*Efhd2^+/+^* (*p* = 0.2421, *g* = 0.9) and Tau_*P301L*_/*Efhd2^–/–^* (*p* = 0.9203, *g* = 1.0). Genotype comparisons showed a large reduction in pSer422 staining at 6 months in Tau_*P301L*_/*Efhd2^–/–^* compared with Tau_*P301L*_/*Efhd2^+/+^* (*p* = 0.9998, *g* = 0.9), and a medium-to-large reduction at 12 months (*p* = 0.4323, *g* = 0.69).

Taken together, these data suggest that *Efhd2* deletion is associated with modest reductions in cortical pSer422 levels at both 6 and 12 months, with larger effect sizes evident in the cortex than in the brainstem. This regional difference may relate to the higher baseline expression of EFhd2 in the cortex (Purohit et al., 2014), potentially making cortical tau phosphorylation more sensitive to EFhd2 loss.

To further characterize the accumulation of pathological tau, we also investigated the difference in age-dependent accumulation of PHF1 between Tau_P301L_/*Efhd2^–/–^* and Tau_P301L_/*Efhd2^+/+^* mice. Physiologically, PHF1 (phosphorylated serine 396 and 404) tau exists at certain levels in normal brains, but it shows marked increase in tauopathies (Greenberg et al., 1992; Otvos et al., 1994; Hasegawa et al., 1996; Kyalu Ngoie Zola et al., 2023). Some studies demonstrated that PHF1 positive tau emerges later than pSer422 labeling NFTs and to a less extent the ghost tangles (Kimura et al., 1996; Augustinack et al., 2002; Moloney et al., 2021). It is widely accepted that PHF1 signal correlates with later stages of pathological tau accumulation (Moloney et al., 2021).

Consistent with the pSer422 pattern, PHF1 immunoreactivity in the brainstem was minimal at 6 months and increased by 12 months (*Figure 5*). Percent stained area was analyzed using two-way ANOVA (Figures 5A–C). The interaction between age and genotype was small [F(1, 18) = 0.5867, *p* = 0.4536, η^2^*p* = 0.03], indicating similar age-related changes across genotypes. A large main effect of age was detected [F(1, 18) = 5.036, *p* = 0.0376, η^2^*p* = 0.22], reflecting overall increases in PHF1 staining from 6 to 12 months. In contrast, the main effect of genotype was negligible [F(1, 18) = 0.0061, *p* = 0.9389, η^2^*p* < 0.01], suggesting that EFhd2 deletion did not meaningfully alter PHF1 levels in the BS.

Holm–Šídák post hoc comparisons were consistent with these effects. Within Tau_P301L_/*Efhd2^+/+^* mice, PHF1 staining increased from 6 to 12 months with a large effect size (*p* = 0.1883, *g* = 1.55). Tau_P301L_/*Efhd2^–/–^* mice showed a medium increase over the same interval (*p* = 0.6593, *g* = 0.57). Between-genotype differences at each age were small to negligible, in line with the nonsignificant and trivial main effect of genotype.

We quantified PHF1 percent stained area in the cortex (CTX) to assess potential genotype- and age-related differences (Figures 5D–F). As shown in Figure 5D, PHF1 immunoreactivity was minimal at 6 months in both Tau_P301L_/*Efhd2^+/+^* and Tau_P301L_/*Efhd2^–/–^* mice. Two-way ANOVA revealed a small interaction between age and genotype (F(1, 18) = 0.8084, *p* = 0.3805, η^2^*p* = 0.04), indicating generally similar age-dependent changes across groups. The main effect of age was medium in magnitude [F(1, 18) = 1.625, *p* = 0.218, η^2^*p* = 0.08], while the main effect of genotype was small [F(1, 18) = 0.7560, *p* = 0.3960, η^2^*p* = 0.04], suggesting modest overall differences in PHF1 staining between genotypes (Figure 5F). Post hoc comparisons reflected these patterns. In Tau_P301L_/*Efhd2^–/–^* mice, PHF1 staining increased from 6 to 12 months with a large effect size (*p* = 0.9559, *g* = 0.96), while Tau_P301L_/*Efhd2^+/+^* mice showed a medium increase over the same interval (*p* = 0.4733, *g* = 0.67). When comparing genotypes at 12 months, PHF1 levels in Tau_P301L_/*Efhd2^–/–^* mice were moderately lower than those in Tau_P301L_/*Efhd2^+/+^* mice (*p* = 0.4733, *g* = 0.6), consistent with a medium-to-large reduction.

Overall, PHF1 accumulation in the cortex increased with age in both genotypes, but the effect sizes suggest a marginal PHF1 reactivity in Tau_P301L_/*Efhd2^–/–^* mice at 12 months. These cortical findings complement the brainstem PHF1 results, supporting the interpretation that *EFhd2* deletion has limited impact on late-stage tau phosphorylation, with regional differences that parallel those observed for pSer422.

In addition to histological assessment of pSer422 and PHF1 tau, we conducted biochemical analysis by western blot on the entire left hemisphere. We showed before the association between EFhd2 and sarkosyl-insoluble high molecular weight tau aggregates (Vega et al., 2008; Ferrer-Acosta et al., 2013b). Moreover, our previous studies provided evidence that this association is not contingent upon phosphorylation states of tau (Vega et al., 2008, 2018, 2019; Ferrer-Acosta et al., 2013b). Therefore, in this study, we used western blotting to biochemically characterize the impact of the absence of EFhd2 on these tau species. First, we measured total tau using the Tau13 antibody in 6 and 12 months as shown in Figure 6. We did not observe a difference in 55 kDa tau species in S1 (soluble tau fraction) among all mice in 6 and 12 months (Figures 6A, B, Tau13). That further supports that deleting the *Efhd2* gene did not influence basal level of tau protein. However, 64 kDa tau that is predominantly detected in P3 fraction, shows a stronger intensity in 12 months compared to 6 months mice (arrowheads in Figures 6A, B). In fact, the 64 kDa tau represents aberrant posttranslationally modified tau that tend to accumulate as higher order multimeric and filamentous species with aging and correlate with behavioral deficit in JNPL3 mice and human tauopathies (Sahara et al., 2002; Berger et al., 2007; Ren and Sahara, 2013).

We quantified the 64 kDa insoluble tau species detected by Tau13 in the P3 fraction to examine age- and genotype-related differences (Figure 6C). Two-way ANOVA revealed a small interaction between age and genotype [F(1, 18) = 0.9325, *p* = 0.3470, η^2^*p* = 0.05], indicating that the pattern of age-related change was generally consistent across groups. A large main effect of age was observed [F(1, 18) = 18.83, *p* = 0.0004, η^2^*p* = 0.51], reflecting a robust increase in the 64 kDa tau species from 6 to 12 months. The main effect of genotype was medium in magnitude [F(1, 18) = 2.417, *p* = 0.1374, η^2^*p* = 0.12], suggesting modest differences between Tau_P301L_/*Efhd2^+/+^* and Tau_P301L_/*Efhd2^–/–^* mice.

Holm–Šídák post hoc comparisons confirmed a large age-related increase in Tau_P301L_/*Efhd2^+/+^* mice, with higher 64 kDa tau at 12 months compared to 6 months (*p* = 0.0067, *g* = 2.2). Tau_P301L_/*Efhd2^–/–^* mice also showed an increase across age, with a large effect size (*p* = 0.0757, *g* = 1.5). Genotype comparisons indicated a large reduction in the 64 kDa tau species at 6 months in Tau_P301L_/*Efhd2^–/–^* relative to Tau_P301L_/*Efhd2^+/+^* (*p* = 0.7065, *g* = 1.2), a pattern that persisted at 12 months (*p* = 0.1238, *g* = 0.8).

Western blot analysis of pSer422 tau revealed no detecTable 55 kDa or 64 kDa pSer422 species in any 6-month-old mice (Figure 6A, pSer422). In contrast, a subset of 12-month-old mice showed a 64 kDa pSer422 band in the P3 fraction (Figure 6B, arrowheads), consistent with prior work in JNPL3 mice showing that higher-molecular-weight pSer422 species are absent at younger ages (Sahara et al., 2002). As illustrated in Figure 6, not all 64 kDa aggregates detected by Tau13 were pSer422-positive, indicating that insoluble tau aggregates comprise heterogeneous phosphorylation states and aggregation intermediates.

To evaluate group differences (Figure 6D), a two-way ANOVA was conducted on the 64 kDa pSer422 signal. The interaction between age and genotype was negligible [F(1, 18) = 0.0094, *p* = 0.9237, η^2^*p* < 0.01], indicating similar age-related patterns across genotypes. A large main effect of age was detected [F(1, 18) = 3.865, *p* = 0.0649, η^2^*p* = 0.18], reflecting an overall increase in 64 kDa pSer422 signal from 6 to 12 months. In contrast, the main effect of genotype was negligible [F(1, 18) = 0.0094, *p* = 0.9237, η^2^*p* < 0.01], and the between-genotype difference at 12 months was likewise negligible (*p* = 0.9858, *g* = 0.06). Together, these findings indicate that while 64 kDa pSer422-positive tau species emerge with age, EFhd2 deletion does not appreciably influence the accumulation of this phosphorylated insoluble tau species.

Western blot analysis of PHF1 detected an age-dependent increase in the 64 kDa insoluble tau species in the P3 fraction (Figures 6A, B, PHF1), whereas PHF1 signal in the S1 fraction remained comparable across mice. These findings are consistent with previous reports showing that 64 kDa PHF1-positive tau is low in younger JNPL3 mice and rises progressively with age and neurodegenerative progression (Sahara et al., 2002; Kametani et al., 2020).

Quantification of the 64 kDa PHF1 band was analyzed using two-way ANOVA. The interaction between age and genotype was small [F(1, 18) = 0.1296, *p* = 0.7230, η^2^*p* = 0.01], indicating similar age-related patterns across genotypes (*Figure 6E*). A large main effect of age was detected [F(1, 18) = 10.29, *p* = 0.0049, η^2^*p* = 0.36], reflecting an overall increase in PHF1 signal from 6 to 12 months. In Tau_P301L_/*Efhd2^+/+^* mice, this increase was supported by a large effect size in the post hoc comparison (*p* = 0.0897, *g* = 1.9). Tau_P301L_/*Efhd2^–/–^* mice likewise showed higher PHF1 signal at 12 months compared to 6 months (*p* = 0.0553), although the absence of detectable PHF1 at 6 months prevented calculation of effect size.

The main effect of genotype was small (F(1, 18) = 0.2214, *p* = 0.6436, η^2^*p* = 0.01), indicating minimal overall differences between Tau_P301L_/*Efhd2^+/+^* and Tau_*P301*L_/*Efhd2^–/–^* mice. Consistent with this, the age-dependent accumulation of PHF1 tau was broadly similar across genotypes.

Taken together, these results support the interpretation that PHF1-positive tau aggregates increase with age in Tau_*P301L*_ mice, as reported previously, and that EFhd2 deletion leads to a slight reduction in PHF1 levels at older ages. This pattern mirrors the reduction observed for pSer422-positive tau in Tau_P301L_/*Efhd2^–/–^* mice and suggests a possible attenuation of late-stage tau aggregation in the absence of EFhd2.

**FIGURE 7:**
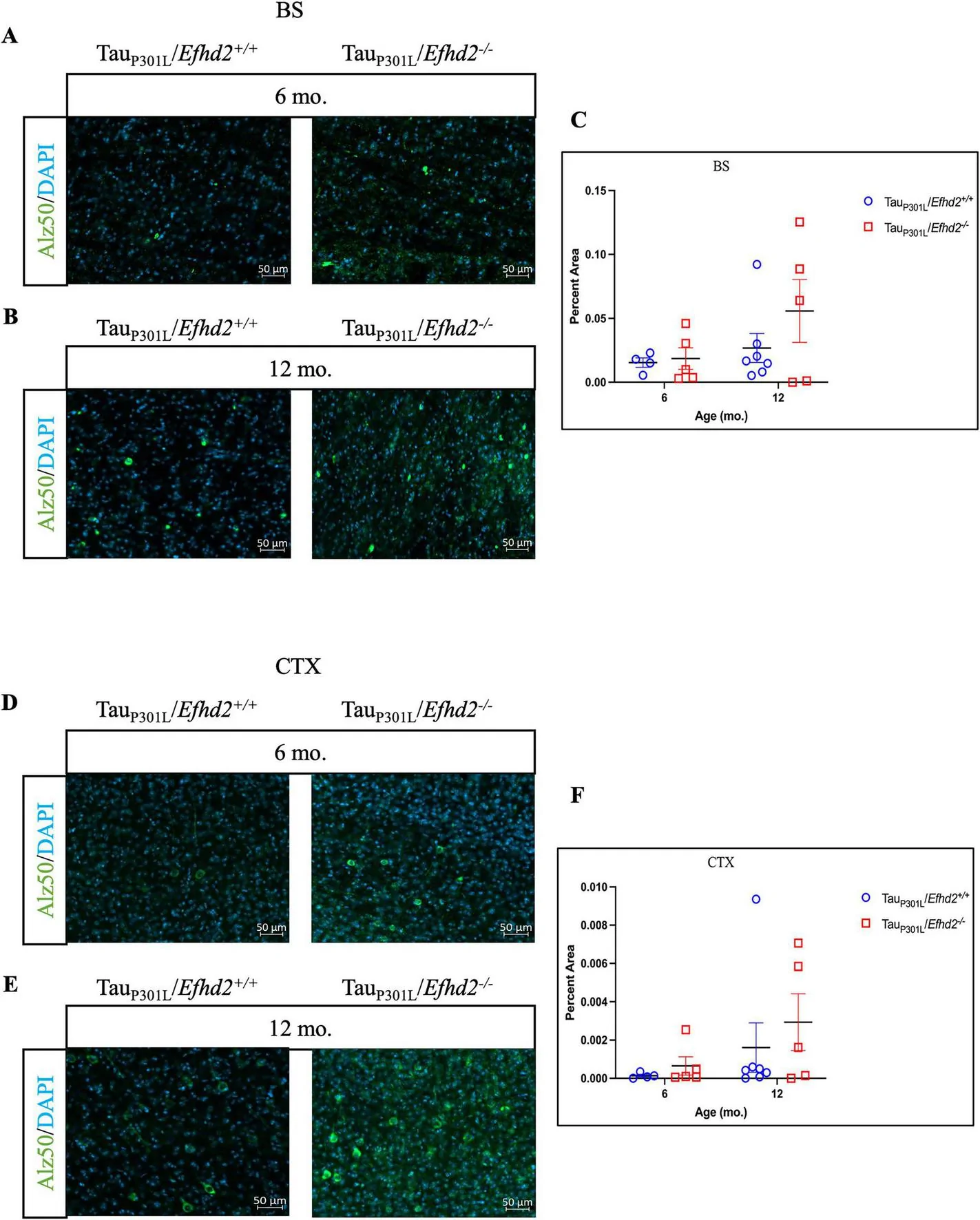
Age- and region-specific changes in Alz50 tau conformation in Tau_*P301L*_ mice with or without *Efhd2*. Right hemispheres from 6- and 12-month-old female Tau_*P301L*_/*Efhd2*^+/+^ and Tau_*P301L*_/*Efhd2*^–/–^ mice (*n* = 4–7/group) were analyzed by immunofluorescence for Alz50 tau (green) and DAPI (blue). (**A, B**) Representative images of Alz50 staining in the brainstem (BS) at 6 and 12 months. **(C)** Quantification of Alz50-positive area in the BS shows age-dependent increases, with a modest elevation in Tau_*P301L*_/*Efhd2*^–/–^ mice. (**D, E**) Representative images of Alz50 staining in the cortex (CTX) at 6 and 12 months. **(F)** Quantification of Alz50-positive area in the CTX indicates age-related changes, with a small-medium increase in Tau_*P301L*_/*Efhd2*^–/–^ mice compared to controls. Data are presented as mean ± SEM. Detailed statistical analyses and corresponding values are provided in the “Results” section.

Lastly, we examined whether EFhd2 deletion influences early pathological tau conformations detected by Alz50. Alz50 recognizes a discontinuous epitope formed when the N-terminus folds over the microtubule-binding domain (Wolozin et al., 1986; Ksiezak-Reding et al., 1988, 1995; Goedert et al., 1991; Carmel et al., 1996), and is widely considered an early marker of pretangle tau aggregates (Guillozet-Bongaarts et al., 2005; Luna-Muñoz et al., 2007; Moloney et al., 2021). Fluorescent staining revealed clear age-related increases in Alz50-positive tau, particularly in the brainstem (BS), where more abundant staining was observed in 12-month-old Tau_*P301L*_/*Efhd2^–/–^* mice compared to 6-month-old mice (Figures 7A, B and Supplementary Figures 4A, B). In contrast, Tau_*P301L*_/*Efhd2^+/+^* mice showed minimal change across age. In both genotypes, Alz50 reactivity was concentrated in neuronal somata with little staining in processes.

Two-way ANOVA showed a modest interaction between age and genotype [F(1, 17) = 0.7526, *p* = 0.3977, η^2^*p* = 0.04], along with a large main effect of age [F(1, 17) = 2.661, *p* = 0.1212, η^2^*p* = 0.14] and a medium main effect of genotype [F(1, 17) = 1.170, *p* = 0.2945, η^2^*p* = 0.06] (Figure 7C). Post hoc comparisons indicated a medium-to-large age-related increase in Alz50 staining in Tau_P301L_/*Efhd2^–/–^* mice (*p* = 0.3338, *g* = 0.9), whereas Tau_P301L_/*Efhd2^+/+^* mice exhibited only a small increase (*p* = 0.8355, *g* = 0.46). At 12 months, Tau_P301L_/*Efhd2^–/–^* mice showed a medium increase in Alz50 staining relative to Tau_P301L_/*Efhd2^+/+^* mice (*p* = 0.4002, *g* = 0.7), while the genotype difference at 6 months was small (*p* = 0.8887, *g* = 0.2).

In the CTX, two-way ANOVA indicated a small age-by-genotype interaction [F(1, 17) = 1.890, *p* = 0.1871, η^2^*p* = 0.01], along with a medium main effect of age [F(1, 17) = 0.3921, *p* = 0.5395, η^2^*p* = 0.13] and a small main effect of genotype [F(1, 17) = 0.8832, *p* = 0.3605, η^2^*p* = 0.01] (Figure 7F). A large effect within-genotype age-related increase was observed in Tau_P301L_/*Efhd2^–/–^* mice (*p* = 0.5686, *g* = 0.93), while Tau_P301L_/*Efhd2^+/+^* mice showed a medium increase (*p* = 0.7712, *g* = 0.52) (Figures 7D, E and Supplementary Figures C, D). Comparisons at 12 months showed a small-to-medium increase in Alz50 staining in Tau_P301L_/*Efhd2^–/–^* relative to Tau_P301L_/*Efhd2^+/+^* mice (*p* = 0.7712, *g* = 0.4) (Figure 7F). At 6 months, Tau_P301L_/*Efhd2^–/–^* mice exhibited moderately higher Alz50 staining (*p* = 0.7773, *g* = 0.63) (Figure 7F).

Overall, EFhd2 deletion was associated with higher levels of Alz50-positive tau in both the brainstem and cortex, particularly at 12 months, with effect sizes in the medium to large range despite non-significant *p*-values. In contrast to the reductions observed in late-stage tau markers such as pSer422 and PHF1, Alz50, which reflects early conformational changes in tau, tended to increase in Tau_P301L_/*Efhd2^–/–^* mice. These contrasting patterns suggest that different pathological tau species may vary in their sensitivity to the absence of EFhd2 and may contribute in distinct ways to the progression of neurodegeneration.

## Discussion

In our previous research, we provided evidence for the association of EFhd2 with pathological tau, especially NFTs, in JNPL3 mouse model and postmortem tauopathies brains (Vega et al., 2008; Ferrer-Acosta et al., 2013b; Vega, 2016). Furthermore, we conducted several *in vitro* studies indicating that EFhd2 modulates the dynamic properties of tau and possibly promotes its aggregation (Vega et al., 2018, 2019). Recently, we reported the unique capacity of recombinant EFhd2 to entangle *in vitro*-formed tau filaments into larger aggregates (Soliman et al., 2024). These aggregates largely resemble NFTs. Hence, we hypothesized that EFhd2 might contribute to tau pathology by enhancing the formation of aggregated pathological tau *in vivo*. In this study, we sought to test this hypothesis by deleting the *Efhd2* gene in Tau_P301L_ expressing mice, thereby investigating to what extent the absence of EFhd2 could impact the formation and progression of tau pathology. Using our novel Tau_P301L_/*Efhd2^–/–^* model, we show, for the first time, that the absence of EFhd2 differentially affects specific pathological tau markers without changing selective brain region vulnerability or the progression of neurodegeneration. The most notable observation in this study is that EFhd2 deletion is associated with shifts in specific tau species, including increased markers of early conformational states and reduced markers of later aggregation stages. These findings suggest that EFhd2 may contribute to the modulation of tau aggregation state rather than acting as a primary driver of tau pathology. The molecular factors that could drive the transition of filamentous tau into mature tangles have eluded the field for a long time. Several studies have identified molecular factors that might contribute to early pathological conformational changes of tau that lead to aberrant oligomerization, which incessantly progresses to tangle formation. Among those factors are PTMs, mutations, and tau-interacting proteins (Van Alstyne et al., 2025). Thus, this study proposes EFhd2 as a potential tau-interacting protein that may govern, at least partly, mature tangle formation.

We first evaluated the outcome of deleting the *Efhd2* gene through longitudinal behavioral assessment to monitor changes in the behavioral phenotype (represented by motor function and nesting). In general, Tau_P301L_ expressing mice develop a pronounced neurodegeneration phenotype around 7 to 8 months in females and beyond 12 months in males (Lewis et al., 2000; Sahara et al., 2002; Lewis and McGowan, 2005). By comparing Tau_P301L_/*Efhd2^+/+^* and Tau_P301L_/*Efhd2^–/–^* mice, neurodegeneration phenotype onset did not greatly change in females and males. Nor did the absence of EFhd2 impacted the magnitude of the decline in the neurodegeneration phenotype. Overall, these findings highlight a more variable longitudinal profile in male Tau_*P301L*_ mice compared with females, consistent with prior reports indicating that male P301L mice typically show a late onset in phenotype – between 12 and 15 months – whereas females exhibit earlier onset (Lewis et al., 2001; Sahara et al., 2002; Lewis and McGowan, 2005). It is important to note, however, that these sex differences primarily reflect differences in the timing of pathology onset rather than differences in the structural features of aggregated tau, which are not known to differ between sexes. Within this context, the variability observed in males in the present study likely reflects an earlier stage of phenotype progression relative to females. Accordingly, the absence of consistent effects of EFhd2 deletion on behavioral outcomes in male mice within the 12-month study window should be interpreted with caution, as later-stage pathological changes were not captured. While EFhd2 deletion did not consistently alter the phenotype trajectory in males, this observation should be interpreted cautiously, given that later stages of pathology were not captured in the current study. These observations warrant further investigation in extended longitudinal studies to determine whether deletion of EFhd2 influences tau-induced behavioral changes during later stages in male JNPL3 mice.

Second, we investigated neuropathological changes due to the absence of EFhd2. We particularly assessed pSer422 and PHF1 as two post-translationally modified tau markers in addition to Alz50 as a conformation tau marker. In fact, numerous studies have shown repeatedly that pSer422 and PHF1 mark later phases of NFTs maturity. Hence, detecting these two markers potentially indicates intermediate/late mature tangles (Kimura et al., 1996; Bussiere et al., 1999; Götz et al., 2001; Augustinack et al., 2002; Deters et al., 2008; Neddens et al., 2018; Moloney et al., 2021). On the other hand, Alz50 recognizes one of the early conformational changes of tau, labeling pretangle forms (Luna-Muñoz et al., 2007; Moloney et al., 2021). Herein, we detected a brain-region specific effect on PHF1 and pSer422 staining areas in Tau_P301L_/*Efhd2^–/–^* mice. We can speculate that the reduction is more prominent in the CTX due to higher EFhd2 expression in forebrain regions than hindbrain regions as reported before (Purohit et al., 2014). In general, the results indicate that EFhd2 absence caused a reduction in late mature tangle markers in both brain regions. Future studies should dissect brain region-specific effects of EFhd2 on the biogenesis of tau aggregates.

Previously, we showed the association of EFhd2 with pathological tau aggregates in sarkosyl-insoluble fraction in AD and Tau_P301L_ mice (Vega et al., 2008; Ferrer-Acosta et al., 2013b). Therefore, we undertook biochemical analysis of pSer422, PHF1, and Tau13 by western blot to assess the influence of EFhd2 absence on the accumulation of sarkosyl-insoluble tau species, especially 64 kDa tau species. The results showed a large reduction in Tau13 detected 64 kDa in the P3 fraction from Tau_P301L_/*Efhd2^–/–^* mice brains when compared with Tau_P301L_/*EFhd2^+/+^*. However, the reduction in pSer422 and PHF1 64 kDa species was small in Tau_P301L_/*Efhd2^–/–^* mice. According to many scholars, 64 kDa and higher molecular weight tau signal advanced events of tau aggregation that follows early phases of pretangle formation and increases with age (Sahara et al., 2002; Berger et al., 2007; Ren and Sahara, 2013). Together, histological assessment of pSer422 and PHF1 (as later tangle markers) along with biochemical assessment of sarkosyl-insoluble tau species highlight the effect of deleting the *Efhd2* gene on accumulation of sarkosyl-insoluble tau species.

We also observed an overall tendency of Alz50 tau to accumulate in Tau_P301L_/*Efhd2^–/–^* mice brains compared to controls. Importantly, statistical analysis, including effect size, endorses the appreciable rise of Alz50-positive tau in Tau_P301L_/*Efhd2^–/–^* mice at 12 months in comparison to Tau_P301L_/*Efhd2^+/+^*. As noted earlier, Alz50 primarily signifies pretangle tau aggregates. It is proposed that the detection of Alz50 decreases as the accumulation of tangles increases because another conformational change leads to N-terminus cleavage and the loss of Alz50 epitope in later fibrils and tangle aggregates (Guillozet-Bongaarts et al., 2005; Moloney et al., 2021). In other words, pathological progression and neurodegeneration might not necessarily correlate with the number of Alz50 positive neurons. In essence, the neuropathological changes induced by deleting the *Efhd2* gene in Tau_P301L_ expressing mice endorse the potential unique capacity of EFhd2 to regulate the transition of early and intermediate filamentous tau into mature tangles.

EFhd2 participates in a broad protein interactome that includes proteins involved in cytoskeletal organization, vesicle trafficking, and protein homeostasis. Therefore, the *in vivo* effects observed in this study are unlikely to be attributable solely to a direct EFhd2–tau interaction. Instead, these findings may reflect the combined influence of EFhd2-associated pathways that indirectly modulate tau aggregation and cellular responses to pathological tau.

For instance, T-cell intracellular antigen 1 (TIA1), an RNA-binding protein that nucleates RNA stress granules (Cruz et al., 2019; Jiang et al., 2019), has the capacity to interact with tau and induce its aberrant folding and neurodegeneration. Using the same approach we used, reducing TIA1 in heterozygous knockout of TIA1 bred with PS19 mice exhibited improved cognitive performance in Y-maze and novel object recognition test (Apicco et al., 2018). Moreover, reducing TIA1 prolonged survival lifespan of PS19 mice. Above all, lower levels of TIA1 led to reduced pretangle oligomeric tau aggregates along with enhanced accumulation of high molecular weight fibrillar tau shown in PS19/*TIA1*^+/–^ mice. Further *in vitro* experiments supported the role of TIA1 in promoting the accumulation of oligomeric pretangle tau forms, and not fibrillar tau (Jiang et al., 2019; Ash et al., 2021). Collectively, these findings indicate that increased TIA1 abundance might exact a toll on neuronal integrity and cognitive performance by sustaining the formation of oligomeric tau and reducing fibrillar tau and NFTs (Maziuk et al., 2018).

Bassoon is another tau-interacting protein that regulates normal synaptic functions and networks (Annamneedi et al., 2018). Recently, bassoon co-purified with seed-competent misfolded and aggregated tau extracted from AD and PS19 brains (Martinez et al., 2022). Consistently, bassoon enhanced tau misfolding and seeding. Moreover, knocking down bassoon in PS19 mice halted tau seeding and propagation. In addition, reducing bassoon levels PS19 mice has rescued behavioral deficit, especially the motor dysfunction (Martinez et al., 2022). Apropos of pathological tau markers, Martinez, et al reported that knocking down bassoon resulted in significant reduction in PHF1 and MC1 (Martinez et al., 2022). It is noteworthy that MC1 antibody recognizes the same tau conformational change detected by Alz50. The results suggest that bassoon stabilizes oligomeric tau and induces a neurotoxic effect.

Recently, more attention has been directed to pretangle oligomeric tau species demonstrating that these early tau aggregates could be the true perpetrator in tau-mediated neurodegeneration. In view of this, TIA1 and bassoon interfere with tau aggregation by enhancing the accumulation of oligomeric tau structures; thus, reducing their levels rescued behavioral deficit and increased higher molecular weight tau forms. Comparatively, our data allude to possible increased accumulation of pretangle oligomeric forms (Alz50 positive) and reduced pSer422 and PHF1 (later tangle markers) upon deleting *Efhd2* gene at old age. Previously, we discovered EFhd2 as a tau-associated protein in sarkosyl-insoluble fraction (Vega et al., 2008). Furthermore, immunogold labeling confirmed the colocalization of EFhd2 and tau in sarkosyl-insoluble tangles (Ferrer-Acosta et al., 2013b). The presented data, together with our previous research, imply that EFhd2 uniquely impacts the biogenesis of pathological tau aggregates by driving the formation of higher order tangle forms.

The absence of robust changes in behavioral outcomes limits conclusions regarding the functional consequences of EFhd2 deletion. While shifts in tau species were observed, it remains unclear whether these molecular differences translate into meaningful effects on neuronal integrity or disease progression. Based on the observed increase in early conformational tau (e.g., Alz50) and reductions in late-stage markers (e.g., pSer422 and PHF1), one could anticipate an accelerated or worsened neurodegenerative phenotype. We speculate that *Efhd2* deletion may induce tau-independent changes that influence behavior, such as alterations in cytoskeletal dynamics, synaptic function, or neuroinflammatory tone, thereby decoupling molecular shifts in tau species from overt behavioral outcomes. Moreover, the Tau_*P301L*_ model presents a robust, age-dependent behavioral deficit and pathological burden. Such conditions may result in a ceiling effect, whereby early aggregation and propagation of pathological tau are already maximized in the Tau_*P301L*_ model. Under these circumstances, the absence of EFhd2 may alter the composition of tau species without producing substantial changes in overall disease progression or behavioral outcomes. Therefore, we should be wary in inferring a neuroprotective role for EFhd2 solely from changes in tau aggregate composition. To distinguish direct effects of EFhd2 on tau biogenesis from its tau-independent biological role, further studies that investigate protracted absence of EFhd2 beyond 12 mo. become necessary. Chiefly, complementary behavioral domains and circuit-level readouts should be assessed in addition probe non-tau pathways affected by *Efhd2* deletion.

Another limitation of the present study is the use of a single tauopathy model. While the Tau_*P301L*_ model provides a well-established system to study tau aggregation and disease progression, it does not fully capture the heterogeneity of human tauopathies. In addition, although female JNPL3 mice develop pathological features earlier than males, the overall characteristics of tau pathology are not fundamentally different between sexes. Nevertheless, because the present pathological analyses were primarily conducted in female mice, the extent to which these findings translate to pathology in males remains to be determined. Therefore, the generalizability of these findings to other models, or pathology in male JNPL3 mice remains uncertain. Future studies will be necessary to evaluate the role of EFhd2 across additional tauopathy models and to determine whether similar effects on tau species distribution are observed.

In conclusion, our findings indicate that *Efhd2* deletion is associated with a shift in tau pathology toward earlier conformational states (higher Alz50) alongside lower late-stage markers (pSer422 and PHF1) in select regions, particularly the cortex, with effect sizes in the small-to-large range but limited evidence of genotype effects on behavior within the observed window. A comprehensive characterization of the Tau_P301L_/*Efhd2^–/–^* model is a critical next step to determine whether these molecular shifts reflect altered progression toward neurofibrillary tangles and how they relate to neurodegeneration over longer intervals and across additional behavioral domains. Future work should incorporate additional conformational tau markers and structural analyses of fibril and tangle architecture and evaluate EFhd2 overexpression to test bidirectional effects on tau aggregate maturation. Given the higher variability observed in males, unbiased proteomic profiling may help identify modifiers that confer resilience or susceptibility. Finally, because EFhd2 operates within a broader protein network, studies should also assess EFhd2-associated proteins as potential contributors to regional and stage-specific modulation of tau pathology.

## Data Availability

The raw data supporting the conclusions of this article will be made available by the authors, without undue reservation.

## Supplementary material

Supplementary Figure 1 Genomic marker background assessment – SNPs and genotyping. **(A)** Illustrates the SNPs associated to either Swiss Webster (SWR), C57BL/6J or Mixed (Het Sw.B6) genomic background. Mice were selected randomly (*n* = 3). The number assigned to each mouse is listed on the graph. The mice maintained a stable homogeneous genomic background. (**B**) PCR-based genotyping. It shows the presence of *Efhd2* in the wild type (*Efhd2^+/+^*) while detection of the Neomycin cassette (*Neo*) verifies the knockout (*Efhd2*^–/–^). In addition, the presence of *MAPT* confirms Tau_P301L_ and its absence indicates Non-Tg (no tau). Positive and negative controls were included in each genotyping cycle for verification. (bp) denotes DNA base pair.

Supplementary Figure 2 Longitudinal change of raw nesting and motor impairment scores. NS and MIS used in computing the combined phenotype score in Figure 2. NS for females (**A**) and males (**B**). MIS for females (**C**) and males (**D**).

Supplementary Figure 3 The deletion of *Efhd2* did not change selective vulnerability of brain regions. Sagittal brain sections of 12 months Tau_P301L_/*Efhd2^+/+^* (**A**) and Tau_P301L_/*Efhd2*^–/–^ (**B**) stained with pSer422. Consistent with the literature, accumulation of tau pathology pervades brainstem, hypothalamus, basal ganglia, and the cortex with minimal accumulation in the hippocampus. The absence of EFhd2 in Tau_P301L_ (JNPL3) mice did not change tau propagation to these brain regions.

Supplementary Figure 4 Low-magnification (zoomed-out) view of Alz50 immunofluorescence shown in Figure 7. Right hemispheres from 6- and 12-month-old female Tau_*P301L*_/Efhd2^+/+^ and Tau_*P301L*_/Efhd2^–/–^ mice (*n* = 4–7 per group) were processed for immunofluorescence analysis of Alz50-positive tau (green) and DAPI (blue). Representative low-magnification images are shown for brainstem (BS; **A, B**) and cortex (CTX; **C, D**). Panels **A** and **C** correspond to 6-month-old mice, and panels **B** and **D** correspond to 12-month-old mice. Scale bar = 500 μm.
